# Caspase-9 Is a Positive Regulator of Osteoblastic Cell Migration Identified by diaPASEF Proteomics

**DOI:** 10.1021/acs.jproteome.3c00641

**Published:** 2024-03-18

**Authors:** Kamila Říhová, Petr Lapčík, Barbora Veselá, Lucia Knopfová, David Potěšil, Jana Pokludová, Jan Šmarda, Eva Matalová, Pavel Bouchal, Petr Beneš

**Affiliations:** † Department of Experimental Biology, Faculty of Science, Masaryk University, Brno 625 00, Czech Republic; ‡ International Clinical Research Center, St. Anne’s University Hospital, Brno 602 00, Czech Republic; § Department of Biochemistry, Faculty of Science, Masaryk University, Brno 625 00, Czech Republic; ∥ Laboratory of Odontogenesis and Osteogenesis, Institute of Animal Physiology and Genetics, Czech Academy of Sciences, Brno 602 00, Czech Republic; ⊥ Proteomics Core Facility, Central European Institute for Technology, Masaryk University, Brno 625 00, Czech Republic; # Department of Physiology, Faculty of Veterinary Medicine, University of Veterinary Sciences, Brno 612 42, Czech Republic

**Keywords:** ABHD2, Caspase 9, diaPASEF, migration, osteoblasts, proteomics

## Abstract

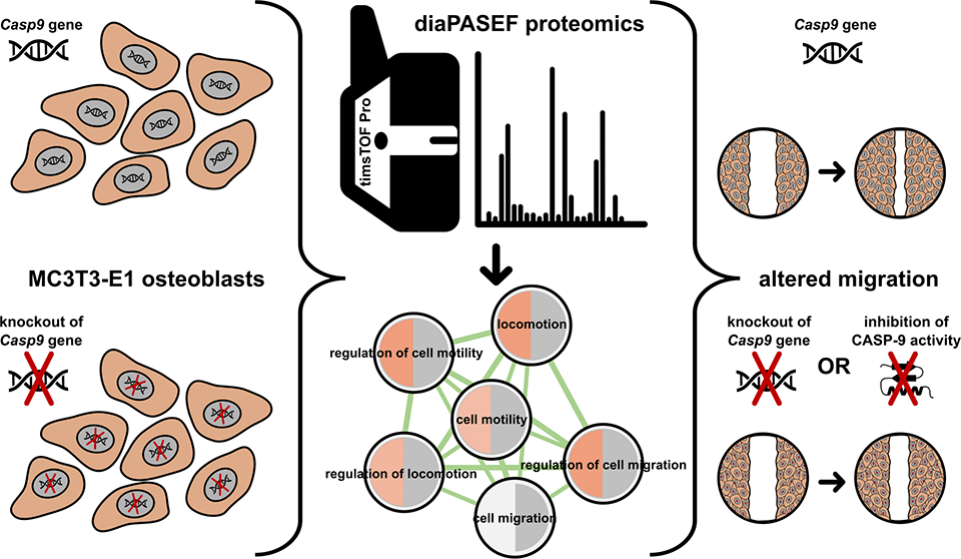

Caspase-9 is traditionally considered the initiator caspase of the intrinsic apoptotic pathway. In the past decade, however, other functions beyond initiation/execution of cell death have been described including cell type-dependent regulation of proliferation, differentiation/maturation, mitochondrial, and endosomal/lysosomal homeostasis. As previous studies revealed nonapoptotic functions of caspases in osteogenesis and bone homeostasis, this study was performed to identify proteins and pathways deregulated by knockout of caspase-9 in mouse MC3T3-E1 osteoblasts. Data-independent acquisition–parallel accumulation serial fragmentation (diaPASEF) proteomics was used to compare protein profiles of control and caspase-9 knockout cells. A total of 7669 protein groups were quantified, and 283 upregulated/141 downregulated protein groups were associated with the caspase-9 knockout phenotype. The deregulated proteins were mainly enriched for those associated with cell migration and motility and DNA replication/repair. Altered migration was confirmed in MC3T3-E1 cells with the genetic and pharmacological inhibition of caspase-9. ABHD2, an established regulator of cell migration, was identified as a possible substrate of caspase-9. We conclude that caspase-9 acts as a modulator of osteoblastic MC3T3-E1 cell migration and, therefore, may be involved in bone remodeling and fracture repair.

## Introduction

Caspases are an evolutionary conserved family of cysteine proteases with well-defined functions in the regulation of cell death and inflammation.^1,2^ More recently, physiological and disease-related functions of various caspases unrelated to cell death execution and immune response have also been described. These include activities in the developing nervous system that affect synaptic plasticity,^3^ axon/dendrite pruning,^4−6^ their outgrowth^7^ and branching,^8^ stem cell activity (self-renewal, differentiation), and thus the regeneration of various tissues.^9−13^ Studies using knockout (KO) models and specific inhibitors have shown that individual caspases are also involved in aging (oxidative stress, DNA damage)^14−16^ and tumorigenesis (both tumor suppressor and promoter functions for individual caspases have been described).^17−19^

We and others have described nonapoptotic functions of caspases in osteogenesis and bone homeostasis as well. Treatment with the inhibitor of caspase-3 (CASP-3) accelerated bone loss in ovariectomized mice,^20^ Bmp4-induced osteoblastic differentiation of MC3T3-E1 is associated with increased activity of caspases-2, -3, and -8,^21^ and pharmacological and genetic inhibition of caspase-8 inhibited differentiation of these cells by reducing osteocalcin expression.^22,23^ Inhibition of other caspases (-1, -7, and -12) modulated bone and hard tissue homeostasis, osteoblastic differentiation and expression of osteogenic and chondrogenic markers in various models *in vivo* and *in vitro*.^24−27^

Caspase-9 (CASP-9) is classically considered the initiator of the intrinsic apoptotic cascade. Its activation occurs in the apoptosome, a protein complex formed in response to the permeabilization of outer mitochondrial membrane.^28,29^ Alternative CASP-9 activation pathways have also been described.^30^ Besides its well-described role in apoptosis, other physiological functions of CASP-9 have been identified, including regulation of myocyte cell differentiation and proliferation,^31^ development of olfactory sensory neurons (axonal projections, synapse formation, neuronal maturation),^32^ mitochondrial homeostasis and reactive oxygen species production,^30^ endosomal sorting and lysosomal biogenesis.^33^

In this study, the function of CASP-9 in MC3T3-E1 osteoblastic cells was investigated using a proteomic approach. Deregulated proteins were enriched for those associated with cell migration/motility. Pharmacological and genetic inhibition of CASP-9 confirmed the altered cell migration of MC3T3-E1 cells. Abhydrolase domain-containing protein 2 (ABHD2), a negative regulator of cell migration,^34^ was identified as a possible substrate of CASP-9.

## Material and Methods

### Cell Cultures and Generation of *Casp9* KO Clones

The MC3T3-E1 osteoblastic cell line was purchased from the European Collection of Authenticated Cell Culture (c.n. 99072810) and cultured in a humidified incubator (37 °C, 5% CO_2_) in MEM Alpha medium (Gibco, USA) with 10% fetal bovine serum (FBS) (Invitrogen, USA), 100 U/mL penicillin, and 100 μg/mL streptomycin (Lonza, Basel). The MC3T3-E1 *Casp9* KO clones were generated by using the CRISPR/Cas9 approach. Guide RNA (gRNA) sequence CTTCACGCGCGACATGATCG was designed by the CRISPOR online tool^35^ and cloned into the pSpCas9(BB)-2A-GFP plasmid as described previously.^36^ Similarly, oligonucleotides comprising the GFP-target sequence were used to derive a control plasmid that was used for the generation of mock-transfected cells.^37^ MC3T3-E1 cells were transfected using Lipofectamine LTX (ThermoFisher Scientific, USA), and a pool of GFP-positive cells was sorted. Next, single-cell colonies were expanded, and the absence of the CASP-9 protein was verified by immunoblotting. Short Ins/Del mutations in the target sequence of genomic DNA isolated from two independent MC3T3-E1 *Casp9* KO clones (A6 and B1) were confirmed by sequencing. Cell cultures with low passage numbers (<15) were used in all experiments.

### Sample Preparation for Proteomics Analysis

Wt, GFP-control (further labeled as mock), and *Casp9* KO MC3T3-E1 cells were seeded (7 × 10^5^) in the growth medium in four biological replicates. The cells were collected after 48 h using a 1 mM EDTA/PBS solution and then lysed in a buffer containing 8 M urea and 0.5 M TEAB (triethylammonium bicarbonate) pH 8.5, sonicated (50 W, 30 × 0.1 s, 30 s pause, 30 × 0.1 s), and incubated on ice for 75 min. Lysates were further centrifuged at 14,000g and 4 °C for 20 min. Protein concentrations in sample supernatants were determined using a RC-DC protein assay kit (Bio-Rad, USA).

### Protein Digestion

Protein digestion was performed using the Filter-Aided Sample Preparation (FASP) method. 50 μg of protein per sample was transferred to the Microcon filter device, 30 kDa cutoff (Millipore, Germany) containing 200 μL of 8 M urea dissolved in 0.5 M TEAB, pH 8.5. Samples were centrifuged at 14,000g and 20 °C for 15 min. 100 μL of 8 M urea and 10 μL of 50 mM tris (2-carboxyethyl) phosphine were added to the filter, and samples were reduced on a thermomixer at 600 rpm and 37 °C for 60 min and centrifuged at 14,000g and 20 °C for 15 min. In the next step, 100 μL of 8 M urea and 5 μL of 200 mM methylmethanethiosulfonate were added to the samples. The samples were alkylated on a thermomixer at 600 rpm and 25 °C for 1 min, stored without stirring in the dark for 20 min, and centrifuged at 14,000g and 20 °C for 15 min. Subsequently, 100 μL of 0.5 M TEAB was added to the filter, and samples were centrifuged at 14,000g and 20 °C for 20 min. The previous step was repeated once. Enzymatic digestion of proteins was initiated by addition of 100 μL of 0.5 M TEAB and 1.67 μL of 1 μg/μL trypsin solution (Promega, USA) dissolved in 50 mM acetic acid (trypsin:cleaved protein ratio was 1:30). The samples were mixed on a thermomixer at 600 rpm and 37 °C for 1 min and digested overnight at 37 °C without shaking. The next day, peptides were eluted by centrifugation at 14,000g and 20 °C for 15 min.

### Peptide Desalting

C18 Silica MicroSpin columns (NestGroup Inc., USA) were used to desalt the peptides prior to mass spectrometry (MS) analysis. The columns were washed twice with 200 μL of 0.1% trifluoracetic acid (TFA) in acetonitrile and centrifuged at 100g and RT for 3 min, which was followed by two washes with 200 μL of 0.1% TFA in water and centrifuged at 300g and RT for 3 min. Columns were left to hydrate for 15 min at RT and centrifuged at 300g and RT for 3 min. Peptide samples were added to the columns and centrifuged at 500g and RT for 3 min. Then, the columns were washed three times with 200 μL of 0.1% TFA in water and centrifuged at 500g and RT for 3 min. The elution was performed by the addition of 200 μL of 0.1% TFA in 50% acetonitrile and centrifugation at 500g and RT for 3 min, which was followed by 200 μL of 0.1% TFA in 80% acetonitrile and centrifugation under the same conditions and the addition of 200 μL of 0.1% TFA in 100% acetonitrile and centrifugation at 500g and RT for 3 min. Eluates were lyophilized in a SpeedVac and stored at −20 °C.

### LC-MS/MS Identification of Peptides in DIA Mode

LC-MS/MS analyses of all peptides were done using nanoElute system (Bruker, USA) connected to a timsTOF Pro spectrometer (Bruker, USA). One column (no trapping column; separation column: Aurora C18, 75 μm ID, 250 mm long, 1.6 μm particles; Ion Opticks, Australia) mode was used on a nanoElute system with default equilibration and sample loading conditions (separation column equilibration: 4 column volumes at 800 bar; sample loading at 800 bar using 2× pick up volume + 2 μL). Concentrated peptides were eluted by a 120 min linear gradient program (flow rate 300 nL/min, 3–30% of mobile phase B; mobile phase A, 0.1% FA in water; mobile phase B, 0.1% FA in acetonitrile) followed by a system wash step at 80% mobile phase B. The analytical column was placed inside the Column Toaster (40 °C; Bruker, USA) and its emitter side was installed into CaptiveSpray ion source (Bruker, USA).

MSn data were acquired using the data-independent acquisition–parallel accumulation serial fragmentation (diaPASEF) approach with a base method *m*/*z* range of 100–1700 and 1/k0 range of 0.6–1.6 V × s × cm^–2^. The Supplementary Data 1 file defines the *m*/*z* 400–1100 precursor range with equal windows size of 26 Th (including 1 Th overlaps) using two steps each PASEF scan and a cycle time of 100 ms locked to 100% duty cycle.

### Processing of LC-MS/MS Data

Quantitative analysis of the LC-MS/MS DIA data was performed in Spectronaut 15.1 (Biognosys, Switzerland) software using the directDIA approach against the *Mus musculus* UniProt/SwissProt database (2021_03, 17,519 sequences, downloaded on 7/29/2021). Precursor q-value cutoff and experiment protein q-value cutoff were set to 0.01. Peptides identified with q-value < 0.01 in at least 4 of 16 analyses were included (q-value percentile 0.25 setting). Fixed modifications were set to Methylthio (C), and variable modifications were set to Acetyl (Protein N-term) and Oxidation (M). Other parameters were set as default. Differential abundance testing was performed using Student’s *t* test in Spectronaut 15.1; proteins with absolute log2 fold change (|log2FC|) > 0.58 and with q-value < 0.05 were considered differentially abundant between the sample groups. An ANOVA test and the visualization of ANOVA significant proteins in a heatmap were performed using Perseus software^38^ version 2.0.11.0.

### Gene Set Enrichment Analysis

GSEA analysis was performed using the WEB-based GEne SeT AnaLysis Toolkit (WebGestalt).^39,40^ This analysis included all identified proteins sorted by ranking metrics computed as negative log2 of the q-value with the sign of the log2-fold change for each comparison. The organism of interest was set to *Mus musculus,* and the method of interest to GSEA. Analysis was performed against the Gene Ontology Biological Process (GO BP) database with minimum number of genes for a category set to 3 and with FDR significance level 0.05. The results were visualized in R Statistical Software version 4.3.1 using the ggplot2 package^41^ version 3.4.4. The Venn diagram was created using the Venny 2.1 tool.^42^

### Enrichment Analysis of Molecular Pathways

Sets of genes encoding proteins that were either statistically (q-value < 0.05) significantly upregulated (log2FC > 0.58) or downregulated (log2FC < −0.58) in both clones against mock were separately submitted to pathway enrichment analysis using g:Profiler tool^44^ that implements Fisher exact test and multiple-test correction to evaluate pathway enrichment. Lists were added as an unordered query. A list of gene names of all proteins identified in our proteomics experiment was used as a landscape for statistical testing. The organism of interest was set to *Mus musculus*. Pathways from Gene Ontology Biological Process (GOBP), Gene Ontology Molecular Function (GOMF), and Gene Ontology Cellular Compartment (GOCC) databases were included. Electronic GO annotations were excluded. The minimal pathway size was set to 15, and the maximum was set to 1500. The results were visualized using the Cytoscape software (version 3.10.)^45^ with the use of EnrichmentMap application (version 3.3.6)^46^ with the FDR q-value cutoff 0.05 and Edge cutoff (Similarity) 0.375.

### Cell Proliferation

3 × 10^4^ of MC3T3-E1 wt, mock, and *Casp9* KO cells were cultured in 6-well plates for 4 days. The cells were counted daily using a CASY cell counter (Roche).

### Cell Migration

Two different methods were used to analyze the cell migration. First, the migration of control and *Casp9* KO MC3T3-E1 cells was monitored using an xCELLigence instrument (Roche, Switzerland) as described previously.^36^ Briefly, CIM-plates 16 with complete growth medium (10% FBS) in the bottom chambers were assembled. Cells were serum starved for 2 h, detached with 1 mM EDTA/PBS, washed with PBS, counted, and plated in serum-free medium in the upper chambers in duplicates at a density of 7.5 × 10^4^ per well. Impedance (displayed as dimensionless parameter cell index) was monitored every 15 min for 8 h. Second, a scratch (wound healing) assay was used to monitor the migration of control and *Casp9* KO MC3T3-E1 cells. The cells were seeded in a 24-well plate at a density of 3 × 10^4^ per well. The cell monolayer was wounded with a sterile pipet tip 72 h after seeding. Subsequently, the fresh medium or medium supplemented with inhibitor/DMSO as a control was added. The cells were photographed every 3 h for 9 or 12 h postwounding using an Olympus IX53 microscope (×40), and cell migration was analyzed by Fiji (NIH, USA) as changes in wound area (%). A wound healing assay was performed subsequently also with wt MC3T3-E1 cells treated with 100 μM CASP-9 inhibitor (218776, Sigma-Aldrich), 100 μM CASP-3/-7 inhibitor (218832, Sigma-Aldrich) or vehicle.

### Inhibition of CASP-9 and CASP-3/-7 Activity

1 × 10^5^ of MC3T3-E1 cells were seeded into a 6-well plate. After 48 h, the cells were treated with 100 μM CASP-9 inhibitor (218776, Sigma-Aldrich, USA) or 100 μM CASP-3/-7 inhibitor (218832, Sigma-Aldrich, USA) for 6 h and then collected using a 1 mM EDTA/PBS solution for immunoblotting.

### Immunoblotting

Cells were lysed, and proteins were resolved by SDS-PAGE and immunoblotted as described previously.^47^ Blots were probed with CASP-9 (#9508, Cell Signaling Technology, USA), cleaved CASP-3 (#9661, Cell Signaling Technology, USA), ABHD2 (14039-1-AP, Proteintech, Germany), ADAM15 (GTX101599, GeneTex, USA), BST-2 (sc-390719, Santa Cruz Biotechnology, USA; 13560-1-AP, Proteintech, Germany; #60066S, Cell Signaling Technology, USA), or α-tubulin (ab7291, Abcam, UK) specific antibodies and horseradish peroxidase-conjugated mouse or rabbit secondary antibodies (Sigma-Aldrich, USA). The signal was developed with a standard ECL procedure using ClarityTM Western ECL Substrate (Bio-Rad, USA).

### qRT-PCR

Total RNA was isolated using the GenElute Total RNA Purification Kit (Sigma-Aldrich, USA) and cDNA was isolated using the QuantiTect RT Kit (Qiagen, Germany). qPCR was performed with the KAPA SYBR Fast Master mix (KAPA Biosystems, USA) with primers spanning exon–exon junctions (Supplementary Data 2) using the LightCycler 480 (Roche, Switzerland). Mouse *Gapdh* was used as the internal control. The qRT-PCR data were analyzed by the ΔΔCt method.

### Immunohistochemistry

Mouse front limbs and heads (CD1 mouse strain) were collected fresh *post-mortem*, and prenatal (E) stage E15 was examined. The samples were obtained in agreement with the recent legislation in the Czech Republic, law 359/2012 Sb., in which there is no specific requirement for *post-mortem* sampling. Histological sections were deparaffinized in xylene and rehydrated in a gradient series of ethanol. Consecutive sections were pretreated in citrate buffer (10 min/98 °C) for antigen retrieval and then incubated with ABHD2 antibody (14039-1-AP, Proteintech, Germany) or antibody specific to cleaved CASP-9 (9509, Cell Signaling Technology, USA) overnight. After treatment with primary antibodies, the samples were exposed to the secondary anti-rabbit antibody Alexa Fluor 488 (Thermo Fisher Scientific) for 40 min at RT. Nuclei were detected by a ProLong Gold Antifade reagent with DAPI (Thermo Fisher Scientific).

### Statistics

Statistical analysis was performed with Prism v8.0.1 (GraphPad Software, La Jolla, CA). All experimental data are presented as mean ± SD and were analyzed with an unpaired *t* test unless stated otherwise.

## Results

### *Casp9* KO Affects the Proteotype of Osteoblastic Cells

**Figure 1:**
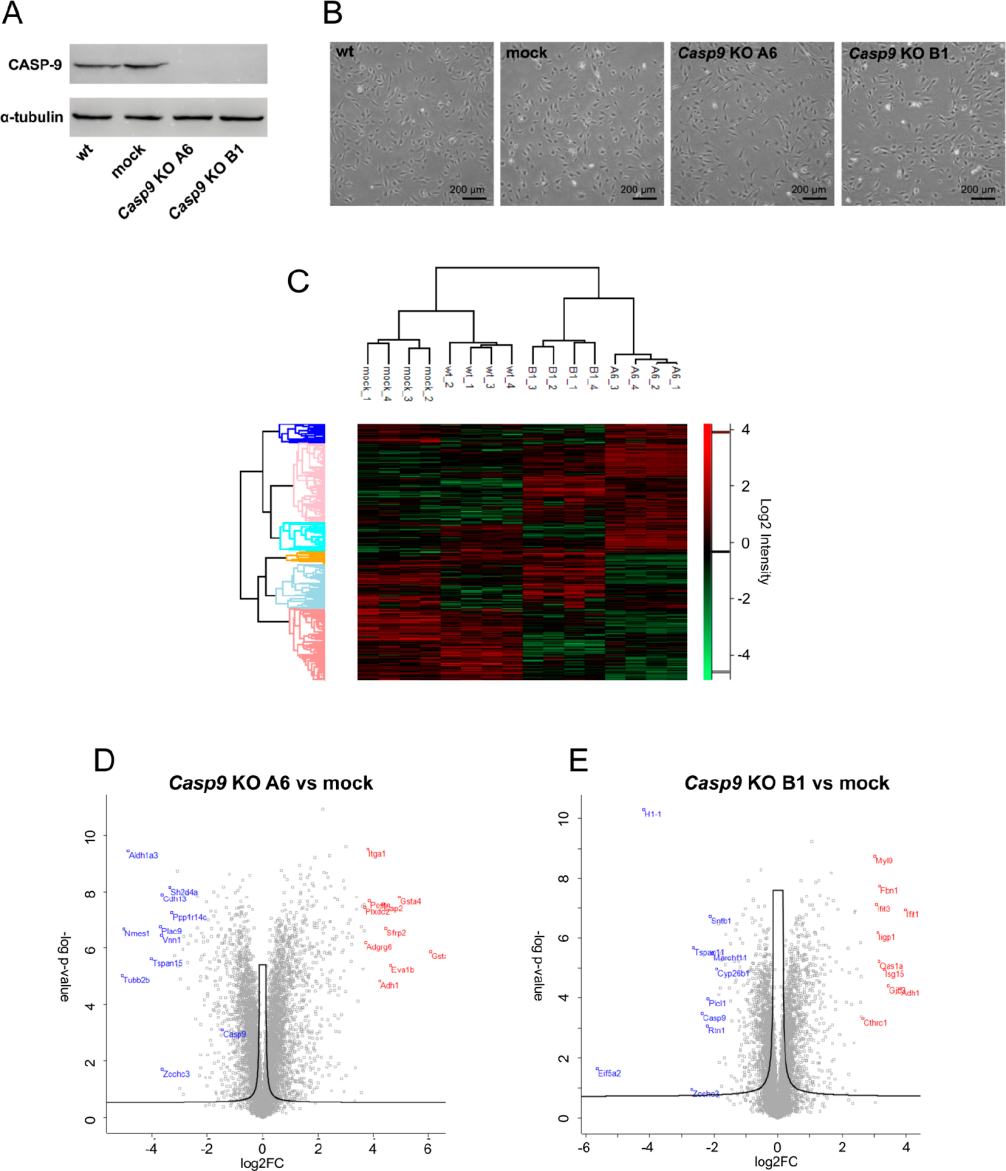
(A) Protein expression of CASP-9 in parental (wt), mock, and *Casp9* KO clones of MC3T3-E1 cell line; α-tubulin was used as a loading control. (B) Morphology of wt, mock, and *Casp9* KO clones of MC3T3-E1 cell line; phase contrast microscopy, total magnification ×40. (C) Heatmap of biological replicates and protein groups clustering of wt, mock, and *Casp9* KO MC3T3-E1 clones according to the sample protein profile. (D) Volcano plot of differential protein abundance analysis between *Casp9* KO A6 clone and mock cells. (E) Volcano plot of differential protein abundance analysis between *Casp9* KO B1 clone and mock cells.

To investigate the function of CASP-9 in osteoblastic cells, two independent MC3T3-E1 *Casp9* KO clones (A6 and B1) were generated using the CRISPR/Cas9 approach. The absence of the CASP-9 protein was confirmed by immunoblotting (Figure 1A) and the presence of a short Ins/Del within the *Casp9* gene was validated by DNA sequencing. Depletion of CASP-9 did not alter cell morphology, as shown in Figure 1B. Next, to identify proteins associated with *Casp9* deficiency in MC3T3-E1 cells, proteome changes in wt, mock, and both *Casp9 KO* clones were evaluated in four biological replicates. Proteins were identified and quantified using LC-MS/MS analysis in the diaPASEF mode.

A total of 7669 protein groups were quantified (FDR < 0.01, for log2 intensity distribution see Supplementary Data 3). Protein levels of CASP-9 were significantly downregulated in the A6 clone (log2FC = −1.30, q-value = 1.25 × 10^–04^) as well as in the B1 clone (log2FC = −2.15, q-value = 2.50 × 10^–06^) compared to the mock cells (Supplementary Data 4) and also compared to the wt cell line (log2FC = −0.75, q-value = 3.95 × 10^–04^ for A6 clone and log2FC = −1.59, q-value = 8.99 × 10^–07^ for B1 clone, Supplementary Data 5, for extracted ion chromatograms, see Supplementary Data 6 and 7). The heatmap (Figure 1C) visualizes the exact clustering of individual biological replicates according to the experimental conditions as well as the clustering of protein groups that are significant in an ANOVA test (q-value < 0.05). Compared to mock cells, *Casp9* KO was associated with significant (q-value < 0.05) upregulation (log2FC > 0.58) and downregulation (log2FC < −0.58) of 1117 and 731 proteins, respectively, in the A6 clone (Figure 1D), and of 476 and 276 proteins in the B1 clone (Figure 1E), respectively. Of these, 283 and 141 proteins were upregulated and downregulated, respectively, in both clones (Supplementary Data 8).

### CASP-9 is Associated with Pathways of Cellular Migration and Adhesion

**Figure 2:**
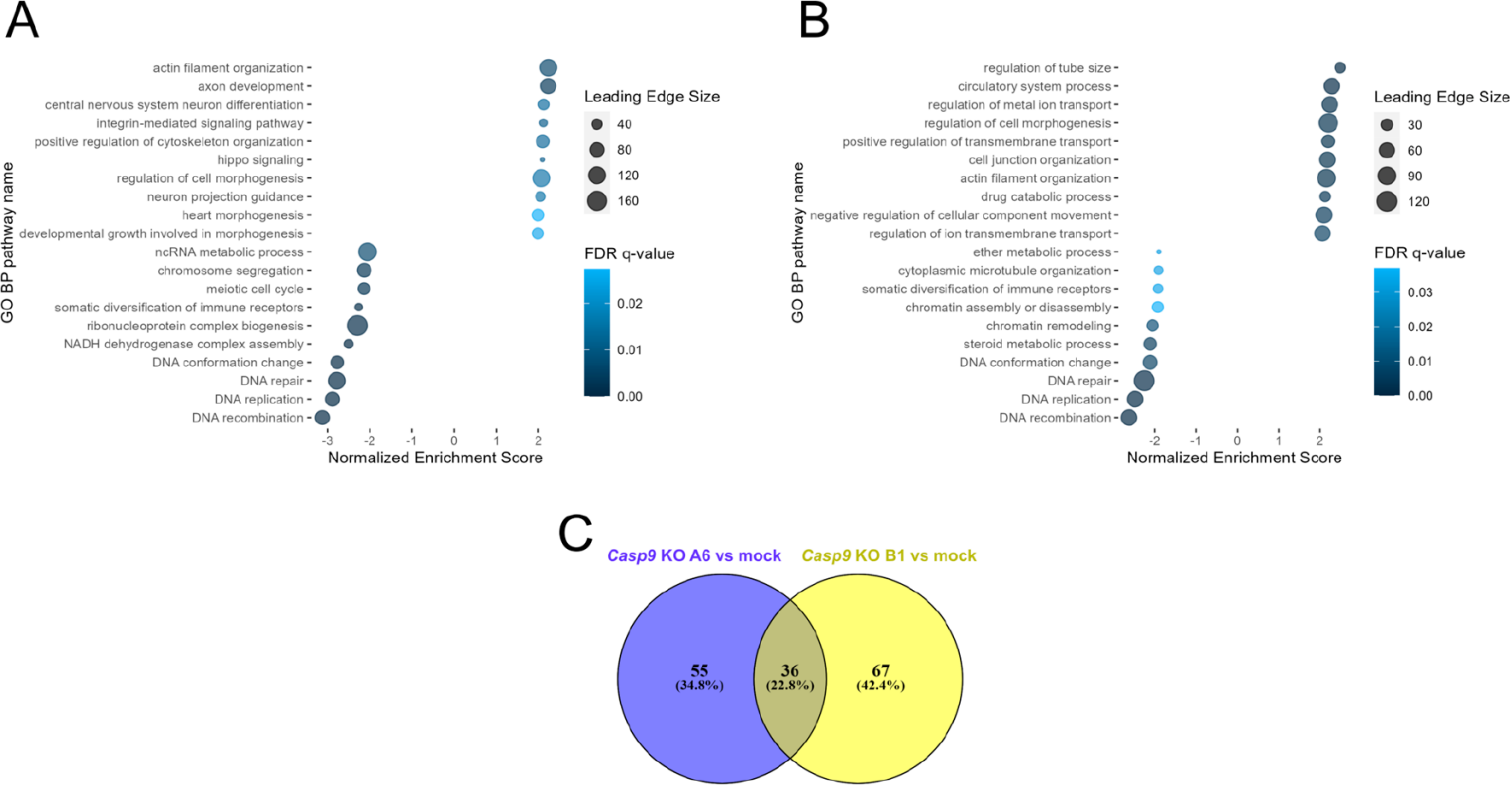
Top 10 significantly positively and negatively enriched GO Biological Process pathways in the WebGestalt GSEA analysis: (A) of the A6 clone proteotype compared to the control mock cell line and (B) of the B1 clone proteotype compared to the control mock cell line. (C) Overlap of significantly enriched GO Biological process pathways in WebGestalt GSEA analysis between A6 vs mock comparison and B1 vs mock comparison.

To describe changes in protein abundances after *Casp9* KO on a proteotype-wide level, GSEA analysis was performed against the Gene Ontology Biological Process database using the WebGestalt tool.^39^*Casp9* KO in A6 and B1 clones compared to mock cells was associated with statistically significant (FDR q-value < 0.05) positive enrichment (normalized enrichment score (NES) > 0) of 71 and 91 GO BP pathways, respectively, and negative enrichment (FDR q-value < 0.05, NES < 0) of 20 and 12 GO BP pathways, respectively (Figure 2A,B, Supplementary Data 9). In total, 30 and 6 GO BP pathways were positively and negatively enriched, respectively, in both clones compared to mock cells (Figure 2C, Supplementary Data 10). The positively enriched pathways in both clones were frequently associated with cytoskeletal organization, morphogenesis, adhesion, and locomotion. On the other hand, DNA replication, recombination and repair were found in negatively enriched pathways.

As a negative control for the *Casp9* KO clones, we compared the proteotypes of wt cells to the mock cells and performed GSEA to define GO BP pathways associated with transfection using the control plasmid. In this comparison, no GO BP pathways were positively enriched, and a total of 35 pathways were negatively enriched (Supplementary Data 9). None of the pathways were negatively enriched in A6 and B1 clones compared with mock cells. These results suggest that the deregulated mechanisms observed in A6 and B1 clones are specific to cells with silenced *Casp9* gene and depend on CASP-9 function.

**Figure 3:**
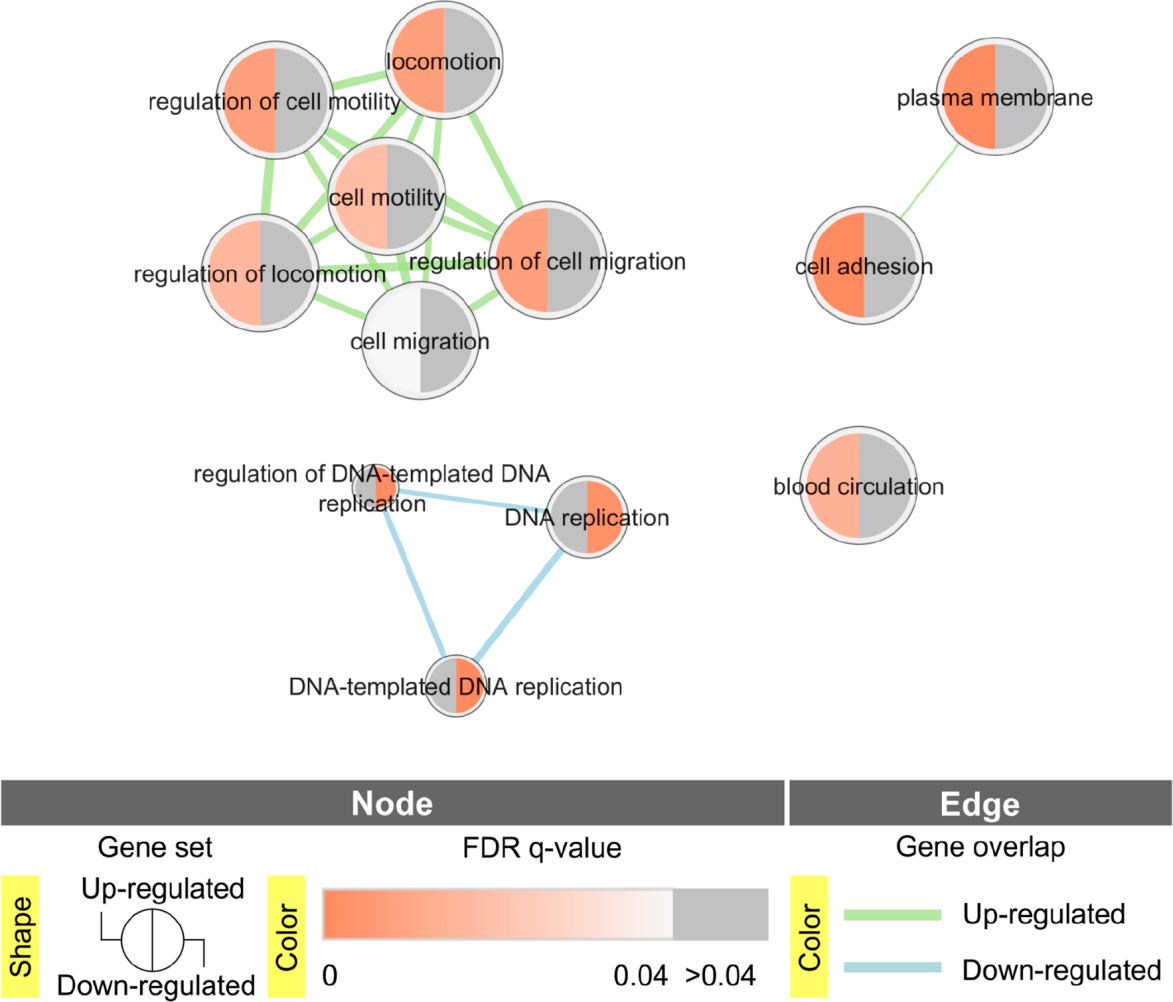
Gene Ontology terms enriched in g:Profiler analysis among up- and downregulated proteins simultaneously in both clones with *Casp9* KO.

Next, an enrichment analysis of Gene Ontology pathways, including Biological Processes (GOBP), Molecular Function (GOMF), and Cellular Compartment (GOCC) terms, was performed using the g:Profiler tool^44^ to define biological pathways consisting of proteins strictly up- or downregulated by *Casp9* KO. These analyses included lists of 283 significantly upregulated or 141 significantly downregulated proteins in both clones simultaneously compared to the mock cell line. Enriched pathways among the upregulated proteins included 8 GOBP and 1 GOCC terms (Figure 3, Supplementary Data 11). These include regulation of cell migration and motility, cell adhesion, and proteins located on the plasma membrane. On the other hand, downregulated proteins are involved in 3 GOBP pathways that participate in DNA replication (Figure 3, Supplementary Data 11).

The pathways associated with cellular migration enriched in GSEA and g:Profiler analyses included 9 negative regulators of cellular migration that were upregulated in both clones compared to mock and wt cell lines (Table 1).

**Table 1:** Proteins Acting as Negative Regulators of Cell Migration Upregulated after *Casp9* KO in Both Clones Compared to Mock and wt Cells^a^

			A6 vs mock		B1 vs mock		A6 vs wt		B1 vs wt	
UniProt ID	Gene name	Protein Description	log2 FC	q-value	log2 FC	q-value	log2 FC	q-value	log2 FC	q-value
O88839	Adam15	**Disintegrin and metalloproteinase domain-containing protein 15**	0.83	1.68 × 10^–03^	0.91	3.94 × 10^–03^	1.77	1.79 × 10^–05^	1.89	5.97 × 10^–05^
P0C605	Prkg1	cGMP-dependent protein kinase 1	0.63	5.35 × 10^–05^	0.66	1.61 × 10^–04^	0.92	2.13 × 10^–05^	0.96	3.49 × 10^–05^
P28828	Ptprm	Receptor-type tyrosine-protein phosphatase mu	1.39	3.98 × 10^–07^	0.65	7.50 × 10^–09^	1.64	1.77 × 10^–08^	0.91	4.56 × 10^–09^
P58771	Tpm1	Tropomyosin alpha-1 chain	1.44	2.12 × 10^–06^	0.65	2.90 × 10^–07^	1.33	2.01 × 10^–06^	0.56	1.63 × 10^–06^
Q08093	Cnn2	Calponin-2	1.10	7.86 × 10^–07^	0.68	8.66 × 10^–07^	1.31	1.80 × 10^–07^	0.92	5.66 × 10^–08^
Q08879	Fbln1	**Fibulin-1**	1.22	5.10 × 10^–11^	1.03	6.90 × 10^–10^	1.11	4.58 × 10^–09^	0.94	2.58 × 10^–08^
Q80U16	Ripor2	Rho family interacting cell polarization regulator 2	2.11	2.61 × 10^–05^	1.39	5.27 × 10^–04^	1.55	2.50 × 10^–05^	0.88	3.49 × 10^–04^
Q8R2Q8	Bst2	**Bone marrow stromal** antigen 2	1.79	3.97 × 10^–05^	2.67	8.00 × 10^–05^	2.77	3.86 × 10^–06^	3.66	1.51 × 10^–05^
Q9QXM0	Abhd2	**Monoacylglycerol lipase ABHD2**	1.37	1.66 × 10^–07^	1.19	1.50 × 10^–07^	1.46	4.80 × 10^–07^	1.31	2.36 × 10^–06^

a Previously identified proteins upregulated in micromass cultures incubated with CASP-9 inhibitor^48^ are in bold.

### CASP-9 Regulates Migration but Not Proliferation of MC3T3-E1 Cells

**Figure 4:**
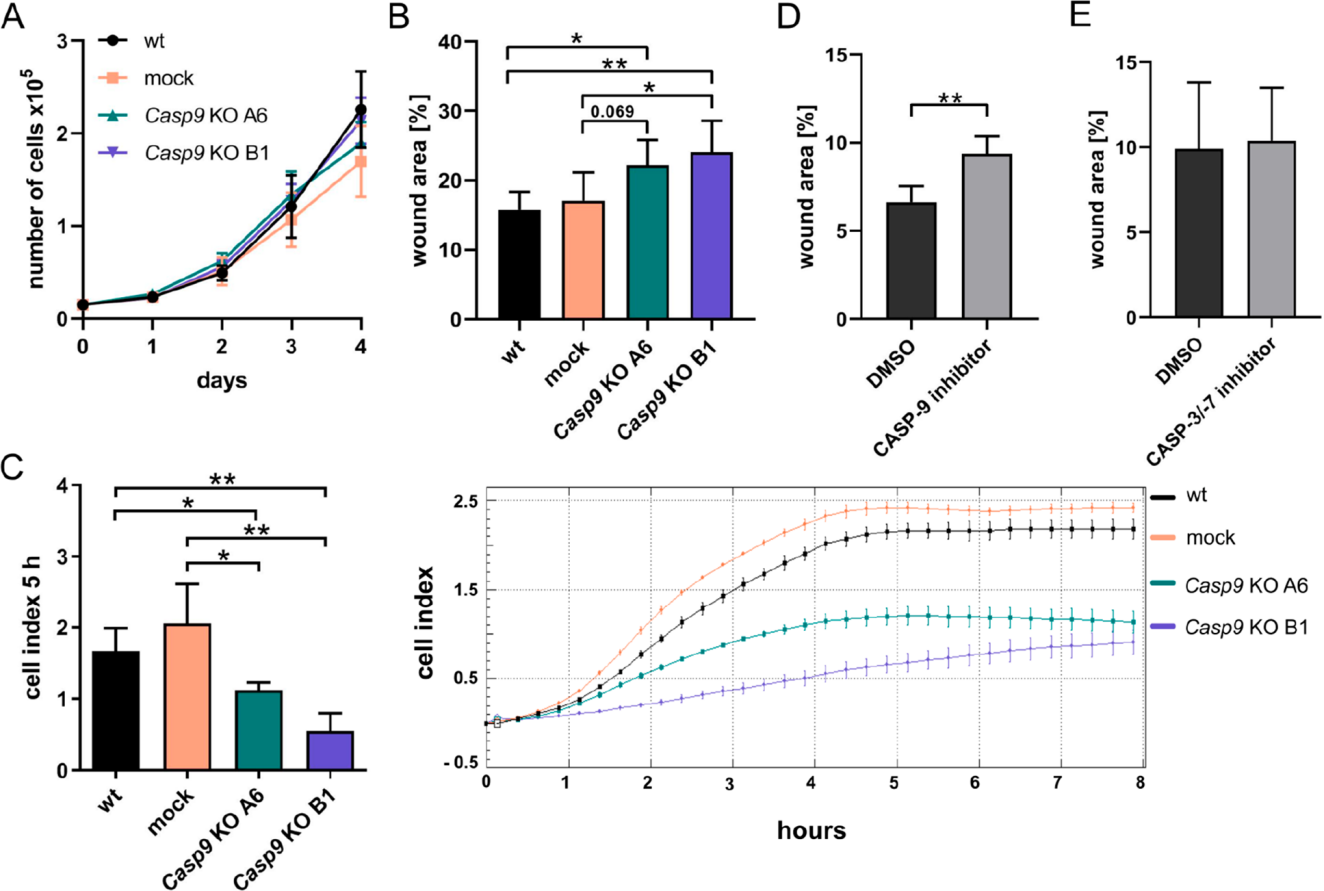
Genetic or pharmacological inhibition of CASP-9 reduces migration but does not affect proliferation of MC3T3-E1 cells. (A) Growth curves of parental (wt), mock, and *Casp9* KO MC3T3-E1 cells. (B, D, E) Migration of wt, mock, and *Casp9* KO MC3T3-E1 cells and wt MC3T3-E1 cells treated with CASP-9 or CASP-3/-7 inhibitor or DMSO as a vehicle control determined using scratch assay. Wound area was analyzed after 12 h (wt, mock, *Casp9* KO clones) or 9 h (CASP-9, CASP-3/-7 inhibitors, DMSO). (C) Migration of parental, mock and *Casp9* KO MC3T3-E1 cells monitored using xCELLigence system. Results of a representative experiment are shown. Cell indexes at 5 h time point interval were compared. Significant differences (*p < 0.05, **p < 0.01) are indicated. Data represents means ± SD from at least three independent experiments.

Proteomic data analysis suggested that CASP-9 may target proteins involved in regulating cell proliferation and migration of MC3T3-E1 cells. Proliferation analysis revealed no difference in the growth rate of *Casp9* KO cells compared to parental and mock cells (Figure 4A). However, the ability of *Casp9* KO cells to migrate was reduced compared to parental and mock cells in both wound healing and transwell/xCELLigence assays (Figure 4B,C). To further confirm the involvement of CASP-9 in regulating the migration of MC3T3-E1 cells, cells were treated with a CASP-9 inhibitor or vehicle, and their migration was analyzed using a wound healing assay. Again, inhibition of CASP-9 enzymatic activity resulted in the reduced migration of MC3T3-E1 cells (Figure 4D). Interestingly, treatment with CASP-3/-7 inhibitor did not affect the migration of MC3T3-E1 cells, suggesting that the migration-promoting role of CASP-9 is not dependent on the activity of downstream caspases (Figure 4E).

### ABHD2 Protein: Possible Substrate of CASP-9

**Figure 5:**
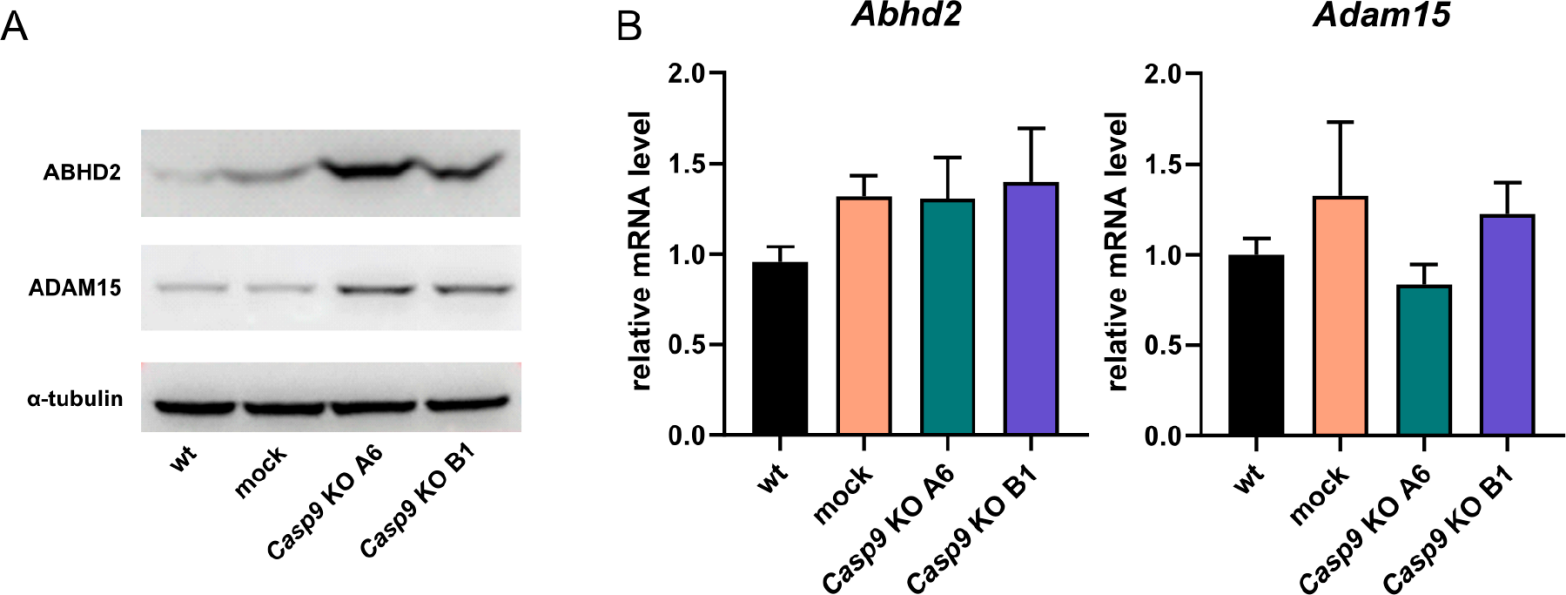
(A) Protein and (B) mRNA levels of ABHD2 and ADAM15 in parental, mock, and *Casp9* KO MC3T3-E1 cells; α-tubulin was used as a loading control for immunoblotting. Data represents means ± SD from at least three independent experiments.

Proteomic analysis revealed 9 possible substrates of CASP-9 that are upregulated in *Casp9* KO cells and were considered negative regulators of cell motility by g:Profiler Gene Ontology pathway analysis (Table 1). After a literature search and screening of available databases,^49,50^ these proteins have not been identified as CASP-9 substrates. Interestingly, four of these proteins (ADAM15, fibulin-1, BST-2, and ABHD2) were found previously to be upregulated in micromass cultures treated with CASP-9 inhibitor by proteomic screen.^48^ Therefore, we further focused our attention on these four proteins. BST-2 was not detected by immunoblotting (Supplementary Data 12) and fibulin-1 has been recently identified as a substrate of CASP-3,^51^ a downstream molecule of CASP-9 in proteolytic cascade, so these two proteins were excluded from further analyses. Increased levels of ABHD2 and ADAM15 proteins were confirmed in both *Casp9* KO clones (Figure 5A). Subsequent qRT-PCR analysis revealed no significant differences in *Abhd2* and *Adam15* expression between the control and *Casp9* KO cells, suggesting that their deregulation occurs at the protein level (Figure 5B).

**Figure 6:**
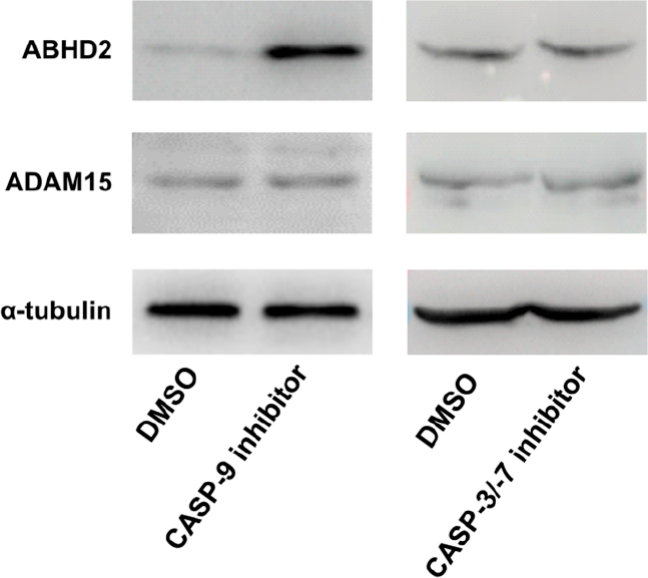
Protein levels of ABHD2 and ADAM15 in MC3T3-E1 cells with inhibited CASP-9 or CASP-3/-7; α-tubulin was used as a loading control.

To confirm the role of CASP-9 in the regulation of ABHD2 and ADAM15, MC3T3-E1 cells were treated with a CASP-9 inhibitor. Subsequent immunoblotting analysis revealed that the ABHD2 protein level increased after CASP-9 inhibition but the ADAM15 protein level remained unchanged (Figure 6). Interestingly, neither ABHD2 nor ADAM15 protein levels were altered by treatment with CASP-3/-7 inhibitor (Figure 6), suggesting that downstream caspases are not involved in the regulation/cleavage of these proteins.

**Figure 7:**
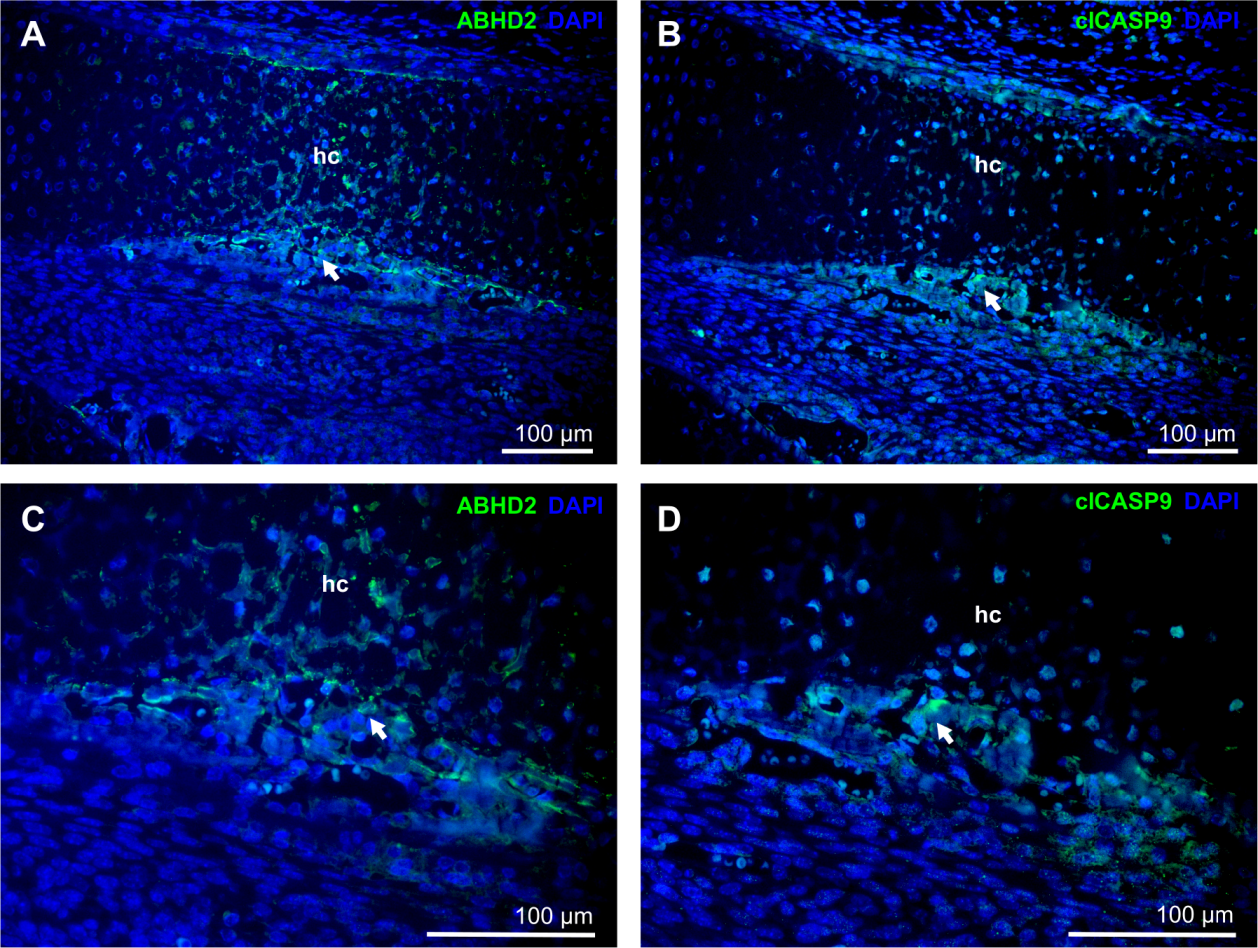
Immunofluorescent staining of ABHD2 (A, C) and cleaved caspase 9 (clCASP9) (B, D) in the developing mouse frontal limb at prenatal day E15. Both proteins were detected in consecutive sections. Positive signal in green (arrows), nuclei counterstained with DAPI (blue); total magnification ×20 (A, B) or ×40 (C, D). hc, hypertrophic cartilage.

ABHD2 is a widely expressed protein known primarily for its function in sperm activation via progesterone signaling.^52,53^ However, its function and expression in osteoblasts during bone development have never been demonstrated. Therefore, to investigate the presence of ABHD2 in osteoblasts *in vivo* and to analyze the colocalization of ABHD2 with cleaved CASP-9, consecutive sections of mouse frontal limbs at prenatal stage E15 were examined by immunofluorescence. In all tested samples, a positive signal of ABHD2 was detected in osteoblasts, and the signal overlapped with that of active CASP-9 (Figure 7). These data confirm the *in vivo* relevance of the results obtained from the cell cultures.

## Discussion

Although caspases are known primarily for their role in various forms of cell death and inflammation,^1,2^ recent studies have identified other physiological and pathophysiological functions of these proteases.^2,54,55^ This also applies for CASP-9 as well. We have previously observed the expression of active CASP-9 in nonapoptotic osteoblasts within the ossification zone of developing long bones.^56^ To the best of our knowledge, the functions of CASP-9 in osteoblasts, beyond the execution of apoptosis, have not been studied yet. We thus performed a proteomic screen to identify possible CASP-9 targets in MC3T3-E1 cells, an osteoblastic cell line derived from mouse calvaria, the common *in vitro* model for osteoblastic lineage. MC3T3-E1 cells with depleted CASP-9 were generated using the CRISPR/Cas9 approach, and their proteome was compared to the proteome of parental/mock-transfected cells. To map the changes in protein abundances associated with CASP-9 depletion, we used the diaPASEF approach that combines peptide separation using trapped ion mobility spectrometry and precursor *m*/*z* window cycling.^57^ diaPASEF provides sensitive peptide detection and data completeness^57^ and we have previously shown this strategy to achieve superior proteome coverage in chondrogenic micromass cultures compared to other commonly used LC-MS/MS-based proteomics workflows.^48^ In the current study, *Casp9* KO significantly affected, namely, proteins associated with cell migration/motility and DNA replication.

Using two different assays, we confirmed that *Casp9* KO results in impaired migration of the MC3T3-E1 cells. Cell migration was also inhibited by an inhibitor of CASP-9 enzymatic activity, suggesting that the proteolytic activity of CASP-9 plays a role. Previous studies have identified caspases, including caspase-1, -3, -8, and -11, as regulators of cell migration in various cells/tissues.^58−65^ Both enzymatic and nonenzymatic functions were involved. However, studies investigating the role of CASP-9 in the regulation of cell migration are rather limited. Inhibition of DRONC, the fly ortholog of CASP-9, affected border cell migration in *Drosophila* ovary.^66^ Other studies that described an association between CASP-9 level/activity and altered cell migration have been published, but in these studies, apoptosis was induced by drug treatment or gene overexpression, thus making the conclusion about direct link between CASP-9 and cell migration impossible.^67−71^

Our proteomic screen identified proteins, described as negative regulators of cell migration, that were upregulated in *Casp9* KO MC3T3-E1 cells and thus represent possible CASP-9 substrates. This study focused on two of them: ABHD2 and ADAM15. ABHD2 is a member of a family of α/β hydrolase fold domain proteins that mediate lipid metabolism and signal transduction. It is ubiquitously expressed protein known for its role in progesterone-mediated activation of sperm, regulation of calcium signaling, lung development and function, monocyte/macrophage recruitment/differentiation/activity, regulation of viral replication, etc.^53,72^ ABHD2 deficiency enhances migration of vascular smooth muscle cells, resulting in intimal hyperplasia in mice.^34^ However, the expression and function of ABHD2 in osteoblasts have never been determined. We found that the ABHD2 protein is expressed in osteoblasts of the developing mouse limb bones, its protein expression colocalizes with that of active CASP-9, and its level is regulated by CASP-9 in MC3T3-E1 osteoblasts. While genetic depletion or pharmacological inhibition of CASP-9 in MC3T3-E1 cells resulted in an increase of ABHD2 protein, activation of CASP-9 has an opposite effect. Moreover, inhibition of downstream CASP-3 has no effect on the ABHD2 protein, suggesting that this effector caspase is not involved. Thus, our results suggest that ABHD2 is a direct target of CASP-9, although we cannot exclude the possibility that other downstream caspases and/or proteases activated by CASP-9 may play a role.

ADAM15 is another protein identified as upregulated in the proteomic screen of *Casp9* KO cells. Using immunoblotting, we detected a higher level of its 75 kDa form that has been reported to correspond to the mature form of this enzyme.^73,74^ However, treatment with CASP-9 inhibitor did not confirm the deregulation of ADAM15. We hypothesize that the alterations in the mature form of ADAM15 are caused by the rather long-term deregulation of CASP-9 in CRISPR clones. Consistent with this hypothesis, a proteomic screen of chondrogenic micromass cultures revealed higher ADAM15 levels after 7 days of treatment with CASP-9 inhibitor.^48^ In MC3T3-E1 cells, however, prolonged treatment with CASP-9 inhibitor significantly reduces their viability, making longer exposure time intervals difficult to achieve. We thus hypothesize that ADAM15 may not be a direct target of CASP-9 but may rather be deregulated or cleaved by other proteases in response to long-term CASP-9 inhibition.

Another upregulated protein in the proteomic screen of *Casp9* KO cells is Bst-2. Bst-2 is a transmembrane protein with putative immunomodulatory functions.^75^ Bst-2 knockout mice have no obvious phenotypic defects but show an altered antiviral response.^76−78^ To the best of our knowledge, no function of Bst-2 in bone homeostasis has been described. To confirm the data obtained by mass spectrometry, we analyzed the expression of Bst-2 in MC3T3-E1 control and *Casp9* KO cells by immunoblotting using three different antibodies. However, in neither case were we able to obtain any reliable signal at a MW corresponding to the mouse Bst-2 protein (30–35 kDa). We are aware that the Bst-2 protein was detected using mass spectrometry, but the success of immunoblotting detection is highly dependent on the quality of the available antibodies. Although antibody detection may be more sensitive than mass spectrometry, the quality and specificity of antibodies are crucial. The question of Bst-2 as a possible substrate of CASP-9 and the physiological relevance of Bst-2 for bone homeostasis remain thus open.

Bone formation, especially during bone remodeling and fracture repair, requires mature osteoblasts to migrate to specific sites in the three-dimensional environment. Understanding this process is necessary as alterations in osteoblast migration and navigation might significantly affect bone development and metabolic bone diseases such as osteoporosis.^79^ Identification of mechanisms that regulate these processes is thus an important prerequisite for the design of targeted therapies.

## Conclusions

We revealed CASP-9 as a modulator of osteoblastic cell migration, and using a proteomic screen, we identified its possible relevant targets. The ABHD2 protein, a known regulator of cell migration, was subsequently validated as a possible CASP-9 substrate using experiments with the genetic and pharmacological inhibition of this protease. These data may indicate a novel nonapoptotic function of CASP-9 in bone remodeling and fracture repair, as the regulation of osteoblastic cell migration is a key component of these physiological processes.

## Data Availability

The mass spectrometry proteomics data have been deposited to the ProteomeXchange Consortium via the PRIDE^80^ partner repository with the data set identifier PXD045703.

## Supporting Information Available

Supplementary Data 1: The *m*/*z* precursor range windows used for diaPASEF. (XLSX)
Supplementary Data 2: Primer sequences used for qPCR. Supplementary Data 3: Histograms of the distribution of protein group log2 intensities in the total proteome experiment. Supplementary Data 6: Extracted ion chromatograms for CASP-9 precursor _SEDLQSLLLR_.2 from total proteome experiment. Supplementary Data 7: Extracted ion chromatograms for CASP-9 precursor _LFFIQAC(Methylthio(C))GGEQK_.2 from total proteome experiment. Supplementary Data 10. The overlap of Gene Ontology Biological Process pathways significantly (FDR q-value < 0.05) positively (NES > 0) or negatively (NES < 0) enriched in GSEA of both *Casp9* KO clones against mock cells. Supplementary Data 12. Western blot images for BST-2 detection (uncropped figures included). Supplementary Data 13–16: Uncropped Western blot images for Figures 1A, 6A, and 7. (PDF)
Supplementary Data 4: Mass spectrometry protein group level data from total proteome experiment for all comparisons of protein levels in MC3T3-E1 *Casp9* KO clones (A6, B1) and wt cell line compared to control mock cell line. (XLSX)
Supplementary Data 5: Mass spectrometry protein group level data from total proteome experiment for all comparisons of protein levels in MC3T3-E1 *Casp9* KO clones (A6, B1) and mock cell line compared to control wt cell line. (XLSX)
Supplementary Data 8: List of protein groups significantly up- and downregulated in both MC3T3-E1 *Casp9* KO clones (A6, B1) compared to control mock cell line. (XLSX)
Supplementary Data 9: Results of GSEA analysis of total proteome data for MC3T3-E1 *Casp9* KO clones (A6, B1) and wt cell line compared to control mock cell line. (XLSX)
Supplementary Data 11: Results of the g:Profiler enrichment analysis of statistically up- and downregulated proteins in both MC3T3-E1 *Casp9* KO clones A6 and B1 compared to control mock cell line. (XLSX)

## References

[ref1] Van Opdenbosch N.; Lamkanfi M. Caspases in Cell Death, Inflammation, and Disease. Immunity 2019, 50 (6), 1352–1364. 10.1016/j.immuni.2019.05.020. 31216460 PMC6611727

[ref2] Shalini S.; Dorstyn L.; Dawar S.; Kumar S. Old, New and Emerging Functions of Caspases. Cell Death Differ. 2015, 22 (4), 526–539. 10.1038/cdd.2014.216. 25526085 PMC4356345

[ref3] Li Z.; Jo J.; Jia J.-M.; Lo S.-C.; Whitcomb D. J.; Jiao S.; Cho K.; Sheng M. Caspase-3 Activation via Mitochondria Is Required for Long-Term Depression and AMPA Receptor Internalization. Cell 2010, 141 (5), 859–871. 10.1016/j.cell.2010.03.053. 20510932 PMC2909748

[ref4] Kuo C. T.; Zhu S.; Younger S.; Jan L. Y.; Jan Y. N. Identification of E2/E3 Ubiquitinating Enzymes and Caspase Activity Regulating Drosophila Sensory Neuron Dendrite Pruning. Neuron 2006, 51 (3), 283–290. 10.1016/j.neuron.2006.07.014. 16880123

[ref5] Williams D. W.; Kondo S.; Krzyzanowska A.; Hiromi Y.; Truman J. W. Local Caspase Activity Directs Engulfment of Dendrites during Pruning. Nat. Neurosci. 2006, 9 (10), 1234–1236. 10.1038/nn1774. 16980964

[ref6] Puhl J. G.; Masino M. A.; Mesce K. A. Necessary, Sufficient and Permissive: A Single Locomotor Command Neuron Important for Intersegmental Coordination. J. Neurosci. Off. J. Soc. Neurosci. 2012, 32 (49), 17646–17657. 10.1523/JNEUROSCI.2249-12.2012. PMC3538829 23223287

[ref7] Westphal D.; Sytnyk V.; Schachner M.; Leshchyns’ka I. Clustering of the Neural Cell Adhesion Molecule (NCAM) at the Neuronal Cell Surface Induces Caspase-8- and −3-Dependent Changes of the Spectrin Meshwork Required for NCAM-Mediated Neurite Outgrowth. J. Biol. Chem. 2010, 285 (53), 42046–42057. 10.1074/jbc.M110.177147. 20961848 PMC3009930

[ref8] Campbell D. S.; Okamoto H. Local Caspase Activation Interacts with Slit-Robo Signaling to Restrict Axonal Arborization. J. Cell Biol. 2013, 203 (4), 657–672. 10.1083/jcb.201303072. 24385488 PMC3840933

[ref9] Bulatovic I.; Ibarra C.; Österholm C.; Wang H.; Beltrán-Rodríguez A.; Varas-Godoy M.; Månsson-Broberg A.; Uhlén P.; Simon A.; Grinnemo K.-H. Sublethal Caspase Activation Promotes Generation of Cardiomyocytes from Embryonic Stem Cells. PloS One 2015, 10 (3), e0120176 10.1371/journal.pone.0120176. 25763592 PMC4357377

[ref10] Janzen V.; Fleming H. E.; Riedt T.; Karlsson G.; Riese M. J.; Lo Celso C.; Reynolds G.; Milne C. D.; Paige C. J.; Karlsson S.; Woo M.; Scadden D. T. Hematopoietic Stem Cell Responsiveness to Exogenous Signals Is Limited by Caspase-3. Cell Stem Cell 2008, 2 (6), 584–594. 10.1016/j.stem.2008.03.012. 18522851 PMC2991117

[ref11] Dawar S.; Shahrin N. H.; Sladojevic N.; D’Andrea R. J.; Dorstyn L.; Hiwase D. K.; Kumar S. Impaired Haematopoietic Stem Cell Differentiation and Enhanced Skewing towards Myeloid Progenitors in Aged Caspase-2-Deficient Mice. Cell Death Dis. 2016, 7 (12), e2509 10.1038/cddis.2016.406. 27906175 PMC5260989

[ref12] Oomman S.; Strahlendorf H.; Dertien J.; Strahlendorf J. Bergmann Glia Utilize Active Caspase-3 for Differentiation. Brain Res. 2006, 1078 (1), 19–34. 10.1016/j.brainres.2006.01.041. 16700096

[ref13] Freimuth J.; Bangen J.-M.; Lambertz D.; Hu W.; Nevzorova Y. A.; Sonntag R.; Gassler N.; Riethmacher D.; Trautwein C.; Liedtke C. Loss of Caspase-8 in Hepatocytes Accelerates the Onset of Liver Regeneration in Mice through Premature Nuclear Factor Kappa B Activation. Hepatol. Baltim. Md 2013, 58 (5), 1779–1789. 10.1002/hep.26538. 23728913

[ref14] Zhang Y.; Padalecki S. S.; Chaudhuri A. R.; De Waal E.; Goins B. A.; Grubbs B.; Ikeno Y.; Richardson A.; Mundy G. R.; Herman B. Caspase-2 Deficiency Enhances Aging-Related Traits in Mice. Mech. Ageing Dev. 2007, 128 (2), 213–221. 10.1016/j.mad.2006.11.030. 17188333 PMC1828128

[ref15] Lopez-Cruzan M.; Herman B. Loss of Caspase-2 Accelerates Age-Dependent Alterations in Mitochondrial Production of Reactive Oxygen Species. Biogerontology 2013, 14 (2), 121–130. 10.1007/s10522-013-9415-x. 23504374 PMC3657345

[ref16] Shalini S.; Dorstyn L.; Wilson C.; Puccini J.; Ho L.; Kumar S. Impaired Antioxidant Defence and Accumulation of Oxidative Stress in Caspase-2-Deficient Mice. Cell Death Differ. 2012, 19 (8), 1370–1380. 10.1038/cdd.2012.13. 22343713 PMC3392626

[ref17] Hu B.; Elinav E.; Huber S.; Booth C. J.; Strowig T.; Jin C.; Eisenbarth S. C.; Flavell R. A. Inflammation-Induced Tumorigenesis in the Colon Is Regulated by Caspase-1 and NLRC4. Proc. Natl. Acad. Sci. U. S. A. 2010, 107 (50), 21635–21640. 10.1073/pnas.1016814108. 21118981 PMC3003083

[ref18] Lamy L.; Ngo V. N.; Emre N. C. T.; Shaffer A. L.; Yang Y.; Tian E.; Nair V.; Kruhlak M. J.; Zingone A.; Landgren O.; Staudt L. M. Control of Autophagic Cell Death by Caspase-10 in Multiple Myeloma. Cancer Cell 2013, 23 (4), 435–449. 10.1016/j.ccr.2013.02.017. 23541952 PMC4059832

[ref19] Krajewska M.; Kim H.; Shin E.; Kennedy S.; Duffy M. J.; Wong Y. F.; Marr D.; Mikolajczyk J.; Shabaik A.; Meinhold-Heerlein I.; Huang X.; Banares S.; Hedayat H.; Reed J. C.; Krajewski S. Tumor-Associated Alterations in Caspase-14 Expression in Epithelial Malignancies. Clin. Cancer Res. Off. J. Am. Assoc. Cancer Res. 2005, 11 (15), 5462–5471. 10.1158/1078-0432.CCR-04-2527. 16061862

[ref20] Miura M.; Chen X.-D.; Allen M. R.; Bi Y.; Gronthos S.; Seo B.-M.; Lakhani S.; Flavell R. A.; Feng X.-H.; Robey P. G.; Young M.; Shi S. A Crucial Role of Caspase-3 in Osteogenic Differentiation of Bone Marrow Stromal Stem Cells. J. Clin. Invest. 2004, 114 (12), 1704–1713. 10.1172/JCI20427. 15599395 PMC535063

[ref21] Mogi M.; Togari A. Activation of Caspases Is Required for Osteoblastic Differentiation. J. Biol. Chem. 2003, 278 (48), 47477–47482. 10.1074/jbc.M307055200. 12954609

[ref22] Kratochvílová A.; Veselá B.; Ledvina V.; Švandová E.; Klepárník K.; Dadáková K.; Beneš P.; Matalová E. Osteogenic Impact of Pro-Apoptotic Caspase Inhibitors in MC3T3-E1 Cells. Sci. Rep. 2020, 10 (1), 7489 10.1038/s41598-020-64294-9. 32366890 PMC7198622

[ref23] Vesela B.; Killinger M.; Rihova K.; Benes P.; Svandová E.; Kratochvilová A.; Trcka F.; Kleparnik K.; Matalova E. Caspase-8 Deficient Osteoblastic Cells Display Alterations in Non-Apoptotic Pathways. Front. Cell Dev. Biol. 2022, 10, 794407 10.3389/fcell.2022.794407. 35372363 PMC8964645

[ref24] Vesela B.; Kratochvilova A.; Svandova E.; Benes P.; Rihova K.; Poliard A.; Matalova E. Caspase-12 Is Present During Craniofacial Development and Participates in Regulation of Osteogenic Markers. Front. Cell Dev. Biol. 2020, 8, 589136 10.3389/fcell.2020.589136. 33178702 PMC7593616

[ref25] Ramesova A.; Vesela B.; Svandova E.; Lesot H.; Matalova E. Caspase-1 Inhibition Impacts the Formation of Chondrogenic Nodules, and the Expression of Markers Related to Osteogenic Differentiation and Lipid Metabolism. Int. J. Mol. Sci. 2021, 22 (17), 9576 10.3390/ijms22179576. 34502478 PMC8431148

[ref26] Svandova E.; Lesot H.; Vanden Berghe T.; Tucker A. S.; Sharpe P. T.; Vandenabeele P.; Matalova E. Non-Apoptotic Functions of Caspase-7 during Osteogenesis. Cell Death Dis. 2014, 5 (8), e1366 10.1038/cddis.2014.330. 25118926 PMC4454305

[ref27] Matalova E.; Lesot H.; Svandova E.; Vanden Berghe T.; Sharpe P. T.; Healy C.; Vandenabeele P.; Tucker A. S. Caspase-7 Participates in Differentiation of Cells Forming Dental Hard Tissues. Dev. Growth Differ. 2013, 55 (5), 615–621. 10.1111/dgd.12066. 23713787

[ref28] Bratton S. B.; Salvesen G. S. Regulation of the Apaf-1-Caspase-9 Apoptosome. J. Cell Sci. 2010, 123 (19), 3209–3214. 10.1242/jcs.073643. 20844150 PMC2939798

[ref29] Li P.; Zhou L.; Zhao T.; Liu X.; Zhang P.; Liu Y.; Zheng X.; Li Q. Caspase-9: Structure, Mechanisms and Clinical Application. Oncotarget 2017, 8 (14), 23996–24008. 10.18632/oncotarget.15098. 28177918 PMC5410359

[ref30] An H.-K.; Chung K. M.; Park H.; Hong J.; Gim J.-E.; Choi H.; Lee Y. W.; Choi J.; Mun J. Y.; Yu S.-W. CASP9 (Caspase 9) Is Essential for Autophagosome Maturation through Regulation of Mitochondrial Homeostasis. Autophagy 2020, 16 (9), 1598–1617. 10.1080/15548627.2019.1695398. 31818185 PMC8386608

[ref31] Murray T. V. A.; McMahon J. M.; Howley B. A.; Stanley A.; Ritter T.; Mohr A.; Zwacka R.; Fearnhead H. O. A Non-Apoptotic Role for Caspase-9 in Muscle Differentiation. J. Cell Sci. 2008, 121 (22), 3786–3793. 10.1242/jcs.024547. 18957517

[ref32] Ohsawa S.; Hamada S.; Kuida K.; Yoshida H.; Igaki T.; Miura M. Maturation of the Olfactory Sensory Neurons by Apaf-1/Caspase-9-Mediated Caspase Activity. Proc. Natl. Acad. Sci. U. S. A. 2010, 107 (30), 13366–13371. 10.1073/pnas.0910488107. 20624980 PMC2922127

[ref33] Han J.; Goldstein L. A.; Hou W.; Watkins S. C.; Rabinowich H. Involvement of CASP9 (Caspase 9) in IGF2R/CI-MPR Endosomal Transport. Autophagy 2021, 17 (6), 1393–1409. 10.1080/15548627.2020.1761742. 32397873 PMC8204962

[ref34] Miyata K.; Oike Y.; Hoshii T.; Maekawa H.; Ogawa H.; Suda T.; Araki K.; Yamamura K. Increase of Smooth Muscle Cell Migration and of Intimal Hyperplasia in Mice Lacking the Alpha/Beta Hydrolase Domain Containing 2 Gene. Biochem. Biophys. Res. Commun. 2005, 329 (1), 296–304. 10.1016/j.bbrc.2005.01.127. 15721306

[ref35] Concordet J.-P.; Haeussler M. CRISPOR: Intuitive Guide Selection for CRISPR/Cas9 Genome Editing Experiments and Screens. Nucleic Acids Res. 2018, 46 (W1), W242–W245. 10.1093/nar/gky354. 29762716 PMC6030908

[ref36] Říhová K.; Dúcka M.; Zambo I. S.; Vymětalová L.; Šrámek M.; Trčka F.; Verner J.; Drápela S.; Fedr R.; Suchánková T.; Pavlatovská B.; Ondroušková E.; Kubelková I.; Zapletalová D.; Tuček Š.; Múdry P.; Krákorová D. A.; Knopfová L.; Šmarda J.; Souček K.; Borsig L.; Beneš P. Transcription Factor C-Myb: Novel Prognostic Factor in Osteosarcoma. Clin. Exp. Metastasis 2022, 39 (2), 375–390. 10.1007/s10585-021-10145-4. 34994868

[ref37] Knopfová L.; Biglieri E.; Volodko N.; Masařík M.; Hermanová M.; Glaus Garzón J. F.; Dúcka M.; Kučírková T.; Souček K.; Šmarda J.; Beneš P.; Borsig L. Transcription Factor C-Myb Inhibits Breast Cancer Lung Metastasis by Suppression of Tumor Cell Seeding. Oncogene 2018, 37 (8), 1020–1030. 10.1038/onc.2017.392. 29084208 PMC6711763

[ref38] Tyanova S.; Temu T.; Sinitcyn P.; Carlson A.; Hein M. Y.; Geiger T.; Mann M.; Cox J. The Perseus Computational Platform for Comprehensive Analysis of (Prote)Omics Data. Nat. Methods 2016, 13 (9), 731–740. 10.1038/nmeth.3901. 27348712

[ref39] Liao Y.; Wang J.; Jaehnig E. J.; Shi Z.; Zhang B. WebGestalt 2019: Gene Set Analysis Toolkit with Revamped UIs and APIs. Nucleic Acids Res. 2019, 47 (W1), W199–W205. 10.1093/nar/gkz401. 31114916 PMC6602449

[ref40] WebGestalt. https://www.webgestalt.org/ (accessed 2024-02-14).

[ref41] Wickham H. Ggplot2: : Elegant Graphics for Data Analysis; Springer-Verlag: New York, 2016. 10.1007/978-3-319-24277-4.

[ref42] Oliveros J. C. Venny. An Interactive Tool for Comparing Lists with Venn’s Diagrams, 2007–2015. https://bioinfogp.cnb.csic.es/tools/venny/index.html (accessed 2024-02-14).

[ref44] Raudvere U.; Kolberg L.; Kuzmin I.; Arak T.; Adler P.; Peterson H.; Vilo J. G:Profiler: A Web Server for Functional Enrichment Analysis and Conversions of Gene Lists (2019 Update). Nucleic Acids Res. 2019, 47 (W1), W191–W198. 10.1093/nar/gkz369. 31066453 PMC6602461

[ref45] Shannon P.; Markiel A.; Ozier O.; Baliga N. S.; Wang J. T.; Ramage D.; Amin N.; Schwikowski B.; Ideker T. Cytoscape: A Software Environment for Integrated Models of Biomolecular Interaction Networks. Genome Res. 2003, 13 (11), 2498–2504. 10.1101/gr.1239303. 14597658 PMC403769

[ref46] Merico D.; Isserlin R.; Stueker O.; Emili A.; Bader G. D. Enrichment Map: A Network-Based Method for Gene-Set Enrichment Visualization and Interpretation. PloS One 2010, 5 (11), e13984 10.1371/journal.pone.0013984. 21085593 PMC2981572

[ref47] Benes P.; Alexova P.; Knopfova L.; Spanova A.; Smarda J. Redox State Alters Anti-Cancer Effects of Wedelolactone. Environ. Mol. Mutagen. 2012, 53 (7), 515–524. 10.1002/em.21712. 22733624

[ref48] Lapcik P.; Vesela B.; Potesil D.; Dadakova K.; Zapletalova M.; Benes P.; Bouchal P.; Matalova E. DiaPASEF Proteotype Analysis Indicates Changes in Cell Growth and Metabolic Switch Induced by Caspase-9 Inhibition in Chondrogenic Cells. Proteomics 2023, 23 (11), e2200408 10.1002/pmic.202200408. 36960851

[ref49] Rawlings N. D.; Barrett A. J.; Thomas P. D.; Huang X.; Bateman A.; Finn R. D. The MEROPS Database of Proteolytic Enzymes, Their Substrates and Inhibitors in 2017 and a Comparison with Peptidases in the PANTHER Database. Nucleic Acids Res. 2018, 46 (D1), D624–D632. 10.1093/nar/gkx1134. 29145643 PMC5753285

[ref50] Fortelny N.; Yang S.; Pavlidis P.; Lange P. F.; Overall C. M. Proteome TopFIND 3.0 with TopFINDer and PathFINDer: Database and Analysis Tools for the Association of Protein Termini to Pre- and Post-Translational Events. Nucleic Acids Res. 2015, 43 (D1), D290–D297. 10.1093/nar/gku1012. 25332401 PMC4383881

[ref51] Araya L. E.; Soni I. V.; Hardy J. A.; Julien O. Deorphanizing Caspase-3 and Caspase-9 Substrates In and Out of Apoptosis with Deep Substrate Profiling. ACS Chem. Biol. 2021, 16 (11), 2280–2296. 10.1021/acschembio.1c00456. 34553588 PMC9116730

[ref52] Miller M. R.; Mannowetz N.; Iavarone A. T.; Safavi R.; Gracheva E. O.; Smith J. F.; Hill R. Z.; Bautista D. M.; Kirichok Y.; Lishko P. V. Unconventional Endocannabinoid Signaling Governs Sperm Activation via the Sex Hormone Progesterone. Science 2016, 352 (6285), 555–559. 10.1126/science.aad6887. 26989199 PMC5373689

[ref53] Lord C. C.; Thomas G.; Brown J. M. Mammalian Alpha Beta Hydrolase Domain (ABHD) Proteins: Lipid Metabolizing Enzymes at the Interface of Cell Signaling and Energy Metabolism. Biochim. Biophys. Acta 2013, 1831 (4), 792–802. 10.1016/j.bbalip.2013.01.002. 23328280 PMC4765316

[ref54] Hollville E.; Deshmukh M. Physiological Functions of Non-Apoptotic Caspase Activity in the Nervous System. Semin. Cell Dev. Biol. 2018, 82, 127–136. 10.1016/j.semcdb.2017.11.037. 29199140 PMC5988915

[ref55] Kuranaga E. Beyond Apoptosis: Caspase Regulatory Mechanisms and Functions in Vivo. Genes Cells Devoted Mol. Cell. Mech. 2012, 17 (2), 83–97. 10.1111/j.1365-2443.2011.01579.x. 22244258

[ref56] Janečková E.; Bíliková P.; Matalová E. Osteogenic Potential of Caspases Related to Endochondral Ossification. J. Histochem. Cytochem. Off. J. Histochem. Soc. 2018, 66 (1), 47–58. 10.1369/0022155417739283. PMC5761947 29091523

[ref57] Meier F.; Brunner A.-D.; Frank M.; Ha A.; Bludau I.; Voytik E.; Kaspar-Schoenefeld S.; Lubeck M.; Raether O.; Bache N.; Aebersold R.; Collins B. C.; Röst H. L.; Mann M. diaPASEF: Parallel Accumulation-Serial Fragmentation Combined with Data-Independent Acquisition. Nat. Methods 2020, 17 (12), 1229–1236. 10.1038/s41592-020-00998-0. 33257825

[ref58] Zhou M.; Liu X.; Li Z.; Huang Q.; Li F.; Li C.-Y. Caspase-3 Regulates the Migration, Invasion and Metastasis of Colon Cancer Cells. Int. J. Cancer 2018, 143 (4), 921–930. 10.1002/ijc.31374. 29524226 PMC6204286

[ref59] Gorelick-Ashkenazi A.; Weiss R.; Sapozhnikov L.; Florentin A.; Tarayrah-Ibraheim L.; Dweik D.; Yacobi-Sharon K.; Arama E. Caspases Maintain Tissue Integrity by an Apoptosis-Independent Inhibition of Cell Migration and Invasion. Nat. Commun. 2018, 9 (1), 2806 10.1038/s41467-018-05204-6. 30022065 PMC6052023

[ref60] Keller N.; Ozmadenci D.; Ichim G.; Stupack D. Caspase-8 Function, and Phosphorylation, in Cell Migration. Semin. Cell Dev. Biol. 2018, 82, 105–117. 10.1016/j.semcdb.2018.01.009. 29410361

[ref61] Ghosh S.; Hegde A.; Ananthan A. S.; Kataria S.; Pincha N.; Dutta A.; Dutta A.; Athreya S.; Khedkar S.; Dey R.; Bhosale A.; Jamora C. Extracellular Caspase-1 Regulates Hair Follicle Stem Cell Migration during Wound-Healing. bioRxiv 2022, 548529 10.1101/548529.

[ref62] Li J.; Brieher W. M.; Scimone M. L.; Kang S. J.; Zhu H.; Yin H.; von Andrian U. H.; Mitchison T.; Yuan J. Caspase-11 Regulates Cell Migration by Promoting Aip1-Cofilin-Mediated Actin Depolymerization. Nat. Cell Biol. 2007, 9 (3), 276–286. 10.1038/ncb1541. 17293856

[ref63] Antonopoulos C.; Cumberbatch M.; Dearman R. J.; Daniel R. J.; Kimber I.; Groves R. W. Functional Caspase-1 Is Required for Langerhans Cell Migration and Optimal Contact Sensitization in Mice. J. Immunol. Baltim. Md 1950 2001, 166 (6), 3672–3677. 10.4049/jimmunol.166.6.3672. 11238606

[ref64] Campano P.; Valdez L.; Guerra L.; Cheng B.; Tsin A. Role of Caspase-8 on Retinal Endothelial Cell Migration and Angiogenesis. Ann. Vasc. Med. Res. 2023, 10.47739/2378-9344/1154. PMC13186181 42164054

[ref65] Su T. T. Non-Apoptotic Roles of Apoptotic Proteases: New Tricks for an Old Dog. Open Biol. 2020, 10 (8), 200130 10.1098/rsob.200130. 32810419 PMC7479936

[ref66] Geisbrecht E. R.; Montell D. J. A Role for Drosophila IAP1-Mediated Caspase Inhibition in Rac-Dependent Cell Migration. Cell 2004, 118 (1), 111–125. 10.1016/j.cell.2004.06.020. 15242648

[ref67] Sun G. G.; Wang Y. D.; Cui D. W.; Cheng Y. J.; Hu W. N. EMP1 Regulates Caspase-9 and VEGFC Expression and Suppresses Prostate Cancer Cell Proliferation and Invasion. Tumour Biol. J. Int. Soc. Oncodevelopmental Biol. Med. 2014, 35 (4), 3455–3462. 10.1007/s13277-013-1456-x. 24338711

[ref68] Tang D.; Geng L.; Ma J. lncRNA PROX1-AS1Mediates the Migration and Invasion of Placental Trophoblast Cells via the miR-211–5p/Caspase-9 Axis. Bioengineered 2021, 12 (1), 4100–4110. 10.1080/21655979.2021.1953213. 34288800 PMC8806442

[ref69] Zhang X.; Zhai T.; Hei Z.; Zhou D.; Jin L.; Han C.; Wang J. Effects of Platycodin D on Apoptosis, Migration, Invasion and Cell Cycle Arrest of Gallbladder Cancer Cells. Oncol. Lett. 2020, 20 (6), 311 10.3892/ol.2020.12174. 33093920 PMC7573877

[ref70] Boonyarat C.; Sangchavee K.; Plekratoke K.; Yenjai C.; Reubroycharoen P.; Kaewamatawong R.; Waiwut P. Candidone Inhibits Migration and Invasion, and Induces Apoptosis in HepG2 Cells. Biol. Pharm. Bull. 2021, 44 (4), 494–500. 10.1248/bpb.b20-00718. 33504737

[ref71] Sun L.; Jin X.; Xie L.; Xu G.; Cui Y.; Chen Z. Swainsonine Represses Glioma Cell Proliferation, Migration and Invasion by Reduction of miR-92a Expression. BMC Cancer 2019, 19 (1), 247 10.1186/s12885-019-5425-7. 30890138 PMC6425678

[ref72] Bononi G.; Tuccinardi T.; Rizzolio F.; Granchi C. α/β-Hydrolase Domain (ABHD) Inhibitors as New Potential Therapeutic Options against Lipid-Related Diseases. J. Med. Chem. 2021, 64 (14), 9759–9785. 10.1021/acs.jmedchem.1c00624. 34213320 PMC8389839

[ref73] Poghosyan Z.; Robbins S. M.; Houslay M. D.; Webster A.; Murphy G.; Edwards D. R. Phosphorylation-Dependent Interactions between ADAM15 Cytoplasmic Domain and Src Family Protein-Tyrosine Kinases. J. Biol. Chem. 2002, 277 (7), 4999–5007. 10.1074/jbc.M107430200. 11741929

[ref74] Pastén-Hidalgo K.; Hernández-Rivas R.; Roa-Espitia A. L.; Sánchez-Gutiérrez M.; Martínez-Pérez F.; Monrroy A. O.; Hernández-González E. O.; Mújica A. Presence, Processing, and Localization of Mouse ADAM15 during Sperm Maturation and the Role of Its Disintegrin Domain during Sperm–Egg Binding. Reproduction 2008, 136 (1), 41–51. 10.1530/REP-07-0300. 18390692

[ref75] Mahauad-Fernandez W. D.; Okeoma C. M. The Role of BST-2/Tetherin in Host Protection and Disease Manifestation. Immun. Inflamm. Dis. 2016, 4 (1), 4–23. 10.1002/iid3.92. 27042298 PMC4768070

[ref76] Swiecki M.; Wang Y.; Gilfillan S.; Lenschow D. J.; Colonna M. Cutting Edge: Paradoxical Roles of BST2/Tetherin in Promoting Type I IFN Response and Viral Infection. J. Immunol. Baltim. Md 1950 2012, 188 (6), 2488–2492. 10.4049/jimmunol.1103145. PMC3522186 22327075

[ref77] Jones P. H.; Mehta H. V.; Maric M.; Roller R. J.; Okeoma C. M. Bone Marrow Stromal Cell Antigen 2 (BST-2) Restricts Mouse Mammary Tumor Virus (MMTV) Replication in Vivo. Retrovirology 2012, 9, 10 10.1186/1742-4690-9-10. 22284121 PMC3283513

[ref78] Li S. X.; Barrett B. S.; Guo K.; Kassiotis G.; Hasenkrug K. J.; Dittmer U.; Gibbert K.; Santiago M. L. Tetherin/BST-2 Promotes Dendritic Cell Activation and Function during Acute Retrovirus Infection. Sci. Rep. 2016, 6, 20425 10.1038/srep20425. 26846717 PMC4742778

[ref79] Thiel A.; Reumann M. K.; Boskey A.; Wischmann J.; von Eisenhart-Rothe R.; Mayer-Kuckuk P. Osteoblast Migration in Vertebrate Bone. Biol. Rev. Camb. Philos. Soc. 2018, 93 (1), 350–363. 10.1111/brv.12345. 28631442 PMC6218945

[ref80] Perez-Riverol Y.; Bai J.; Bandla C.; García-Seisdedos D.; Hewapathirana S.; Kamatchinathan S.; Kundu D. J.; Prakash A.; Frericks-Zipper A.; Eisenacher M.; Walzer M.; Wang S.; Brazma A.; Vizcaíno J. A. The PRIDE Database Resources in 2022: A Hub for Mass Spectrometry-Based Proteomics Evidences. Nucleic Acids Res. 2022, 50 (D1), D543–D552. 10.1093/nar/gkab1038. 34723319 PMC8728295

