# Supplementary material for: Caspase-9 Is a Positive Regulator of Osteoblastic Cell Migration Identified by diaPASEF Proteomics
Source: J Proteome Res. 2024 Mar 18;23(8):2999–3011. doi: 10.1021/acs.jproteome.3c00641 (PMC11301665; doi:10.1021/acs.jproteome.3c00641)

Caspase-9 is a positive regulator of osteoblastic cell migration identified by diaPASEF proteomics

Kamila Říhová[1,2], Petr Lapčík[3], Barbora Veselá[4], Lucia Knopfová[1,2], David Potěšil[5], Jana Pokludová[1,2], Jan Šmarda[1], Eva Matalová[4,6], Pavel Bouchal[3], Petr Beneš[1,2]*

[1] Department of Experimental Biology, Faculty of Science, Masaryk University, Brno, Czech Republic

[2] International Clinical Research Center, St. Anne's University Hospital, Brno, Czech Republic

[3] Department of Biochemistry, Faculty of Science, Masaryk University, Brno, Czech Republic

[4] Laboratory of Odontogenesis and Osteogenesis, Institute of Animal Physiology and Genetics, Czech Academy of Sciences, Brno, Czech Republic

[5] Proteomics Core Facility, Central European Institute for Technology, Masaryk University, Brno, Czech Republic

[6] Department of Physiology, Faculty of Veterinary Medicine, University of Veterinary Sciences, Brno, Czech Republic

* **Corresponding author**:

Petr Benes, PhD, Department of Experimental Biology, Faculty of Science, Masaryk University, Kamenice 5, Brno, 625 00 Czech Republic, pbenes@sci.muni.cz

Table of contents

Supplementary Data 1. The m/z precursor range windows used for diaPASEF (XLSX, separate file)

Supplementary Data 2. Table of primers used for qPCR

Supplementary Data 3. Histograms of the distribution of protein group log2 intensities in the total proteome experiment

Supplementary Data 4. Mass spectrometry protein group level data from total proteome experiment for all comparisons of protein levels in MC3T3-E1 Casp9 KO clones (A6, B1) and wt cell line compared to control mock cell line (XLSX, separate file)

Supplementary Data 5. Mass spectrometry protein group level data from total proteome experiment for all comparisons of protein levels in MC3T3-E1 Casp9 KO clones (A6, B1) and mock cell line compared to control wt cell line (XLSX, separate file)

Supplementary Data 6. Extracted ion chromatograms for CASP-9 precursor **_SEDLQSLLLR_.2** from total proteome experiment

Supplementary Data 7. Extracted ion chromatograms for CASP-9 precursor _LFFIQAC(Methylthio(C))GGEQK_.2 from total proteome experiment

Supplementary Data 8. List of protein groups significantly up- and down-regulated in both MC3T3-E1 Casp9 KO clones (A6, B1) compared to control mock cell line (XLSX, separate file)

Supplementary Data 9. Results of GSEA analysis of total proteome data for MC3T3-E1 Casp9 KO clones (A6, B1) and wt cell line compared to control mock cell line (XLSX, separate file)

Supplementary Data 10. The overlap of Gene Ontology Biological Process pathways significantly (FDR q-value < 0.05) positively (NES > 0) or negatively (NES < 0) enriched in GSEA of both *Casp9* KO clones against mock cells (PDF)

Supplementary Data 11. Results of the g:Profiler enrichment analysis of statistically up- and down-regulated proteins in both MC3T3-E1 Casp9 KO clones A6 and B1 compared to control mock cell line (XLSX, separate file)

Supplementary Data 12. Western blot images for BST-2 detection (uncropped figures included) (PDF)

Supplementary Data 13. Uncropped western blot images for Figure 1A

Supplementary Data 14. Uncropped western blot images for Figure 6A

Supplementary Data 15. Uncropped western blot images for Figure 7 (CASP-9 inhibitor)

Supplementary Data 16. Uncropped western blot images for Figure 7 (CASP-3/-7 inhibitor)

Supplementary Data 2. Table of primers used for qPCR

Primer	sequence 5’-3’
Abhd2_fw	CTGGAATTGCCAACCACAGC
Abhd2_rev	AATTCCCACGTGCAGCCATA
Adam15_fw	CTGGACCTGGGCAATGATGT
Adam15_rev	CTGCTGTCACAGACCCCAT
Gapdh_fw	ACTTGGCAGGTTTCTCCAGG
Gapdh_rev	TCATGACCACAGTCCATGCC

Supplementary Data 3. Histograms of the distribution of protein group log2 intensities in the total proteome experiment

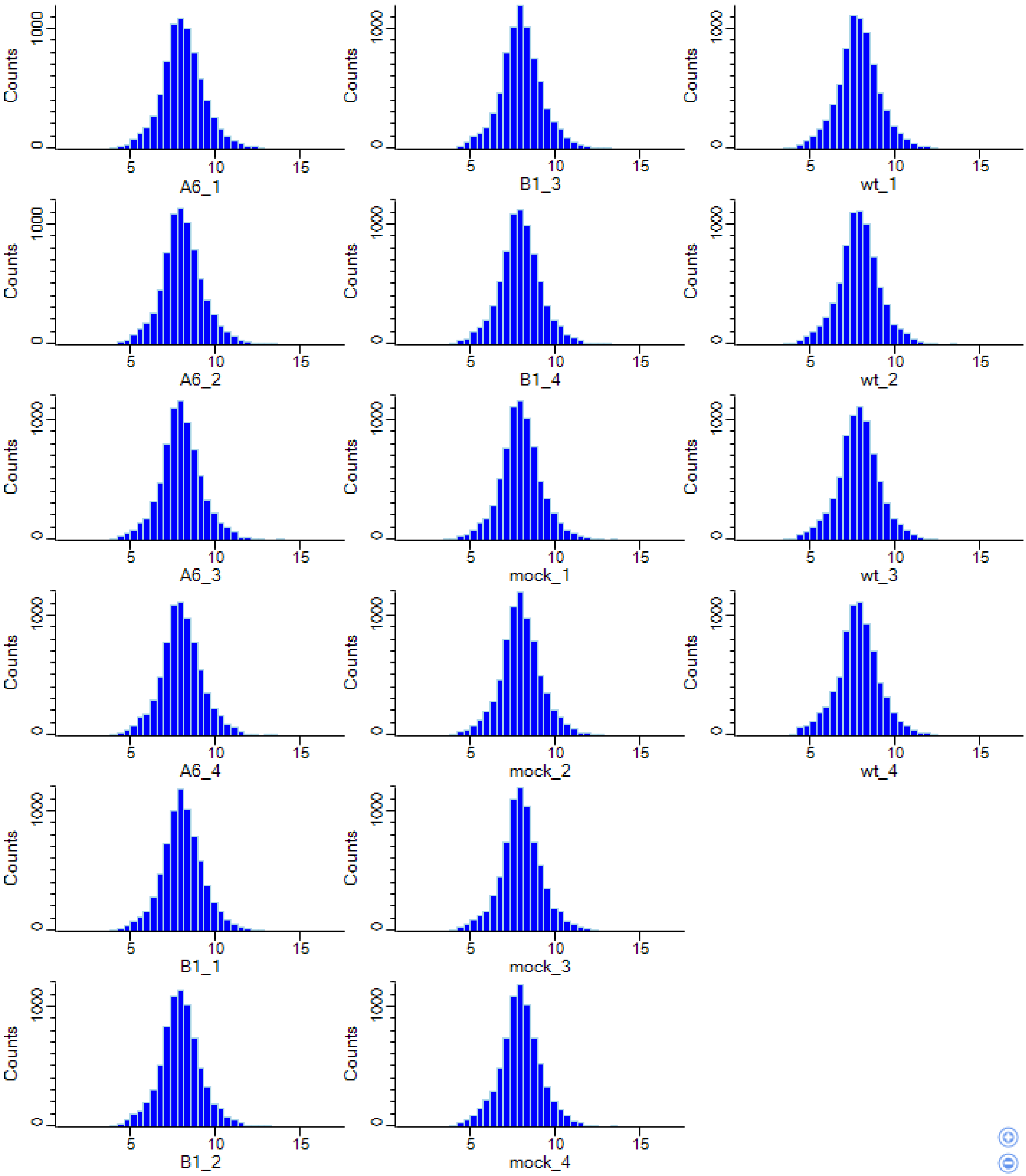

Supplementary Data 6. Extracted ion chromatograms for CASP-9 precursor **_SEDLQSLLLR_.2** from total proteome experiment

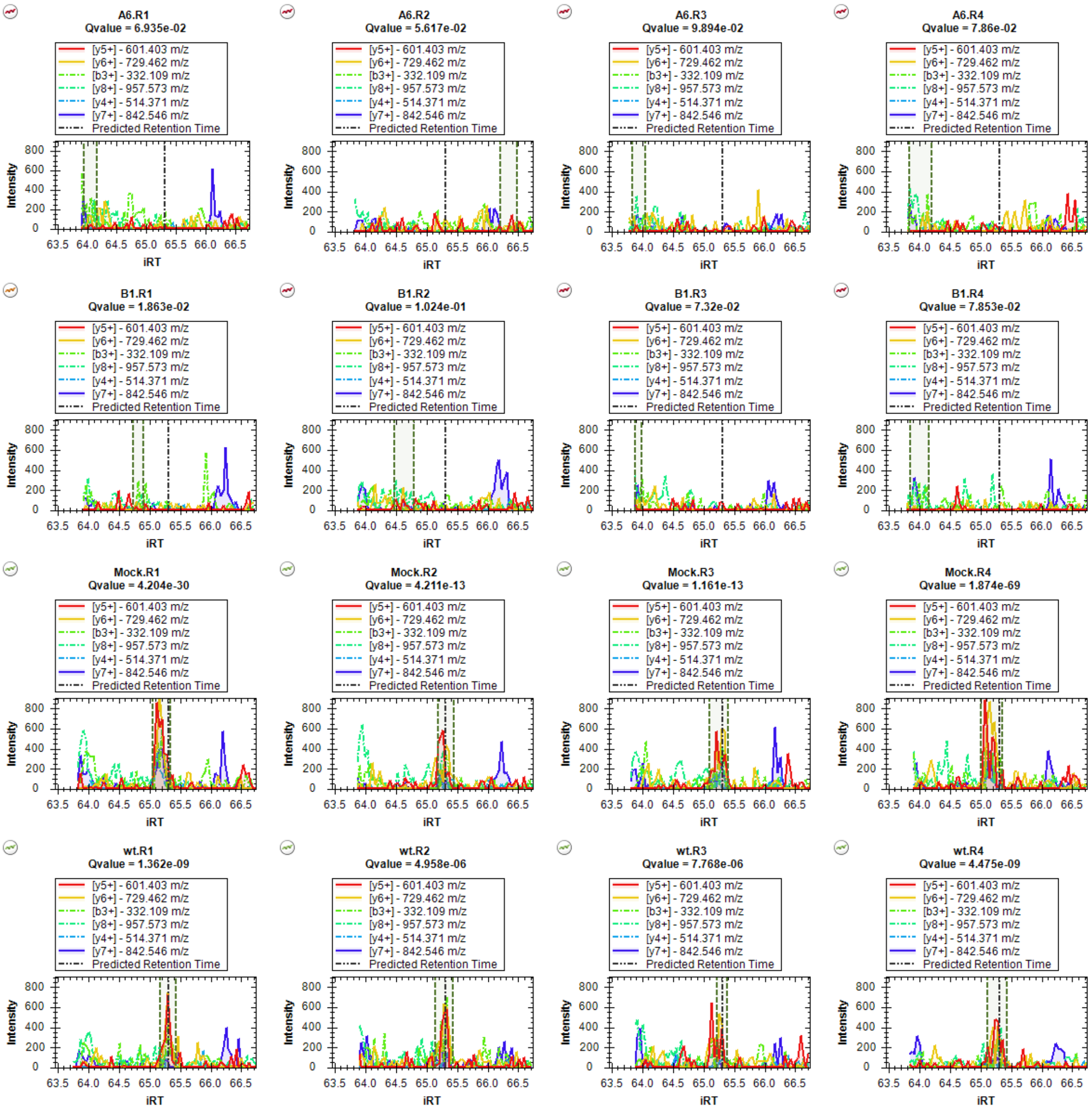

Supplementary Data 7. Extracted ion chromatograms for CASP-9 precursor **_LFFIQAC(Methylthio(C))GGEQK_.2** from total proteome experiment

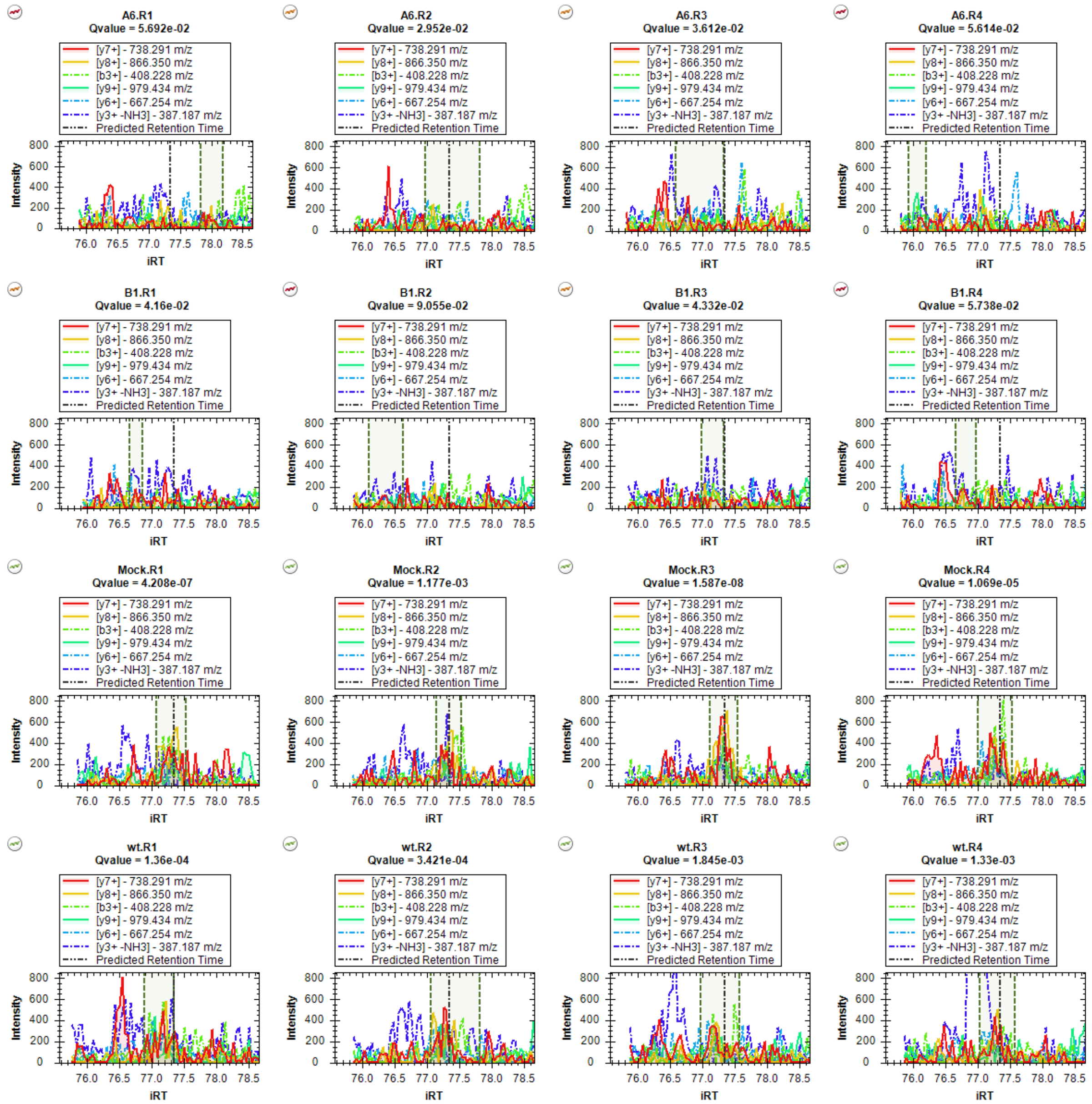

Supplementary Data 10. The overlap of Gene Ontology Biological Process pathways significantly (FDR q-value < 0.05) positively (NES > 0) or negatively (NES < 0) enriched in GSEA of both *Casp9* KO clones against mock cells

GO ID	Pathway name	A6 vs. mock		B1 vs. mock	
		NES	FDR q-value	NES	FDR q-value
GO:0007015	actin filament organization	2.24	0.007	2.15	0.003
GO:0061564	axon development	2.24	0.003	1.90	0.018
GO:0007229	integrin-mediated signaling pathway	2.12	0.011	1.92	0.018
GO:0022604	regulation of cell morphogenesis	2.07	0.016	2.20	0.003
GO:0060560	developmental growth involved in morphogenesis	1.99	0.026	1.91	0.018
GO:0002011	morphogenesis of an epithelial sheet	1.96	0.024	1.73	0.041
GO:1902903	regulation of supramolecular fiber organization	1.93	0.027	1.88	0.019
GO:0031532	actin cytoskeleton reorganization	1.89	0.035	1.83	0.025
GO:0006090	pyruvate metabolic process	1.88	0.034	1.78	0.030
GO:0035150	regulation of tube size	1.88	0.033	2.49	0.000
GO:0034764	positive regulation of transmembrane transport	1.88	0.033	2.19	0.003
GO:0051668	localization within membrane	1.87	0.032	1.90	0.017
GO:0031589	cell-substrate adhesion	1.85	0.038	2.04	0.007
GO:0042737	drug catabolic process	1.83	0.039	2.12	0.004
GO:0099504	synaptic vesicle cycle	1.82	0.040	1.83	0.024
GO:0033627	cell adhesion mediated by integrin	1.81	0.038	1.69	0.045
GO:0099177	regulation of trans-synaptic signaling	1.81	0.038	1.73	0.040
GO:0031346	positive regulation of cell projection organization	1.81	0.039	1.67	0.049
GO:0031032	actomyosin structure organization	1.80	0.042	2.05	0.006
GO:0007264	small GTPase mediated signal transduction	1.78	0.046	1.84	0.023
GO:0051051	negative regulation of transport	1.77	0.045	1.96	0.015
GO:0006520	cellular amino acid metabolic process	1.76	0.046	1.71	0.043
GO:0051271	negative regulation of cellular component movement	1.76	0.045	2.09	0.005
GO:0009612	response to mechanical stimulus	1.76	0.044	1.91	0.018
GO:0043087	regulation of GTPase activity	1.75	0.043	1.82	0.025
GO:0034330	cell junction organization	1.75	0.043	2.18	0.002
GO:0071800	podosome assembly	1.73	0.046	1.86	0.020
GO:0040013	negative regulation of locomotion	1.72	0.047	2.05	0.007
GO:0003013	circulatory system process	1.71	0.049	2.29	0.001
GO:0043254	regulation of protein complex assembly	1.71	0.049	1.67	0.049
GO:0002200	somatic diversification of immune receptors	-2.26	0.001	-1.92	0.034
GO:0010257	NADH dehydrogenase complex assembly	-2.50	0.000	-1.86	0.046
GO:0071103	DNA conformation change	-2.77	0.000	-2.12	0.007
GO:0006281	DNA repair	-2.78	0.000	-2.26	0.001
GO:0006260	DNA replication	-2.88	0.000	-2.48	0.000
GO:0006310	DNA recombination	-3.12	0.000	-2.63	0.000

Supplementary Data 12. Western blot images for BST-2 protein expression in parental, mock and *Casp9* KO clones of MC3T3-E1 cell line (uncropped figures included)

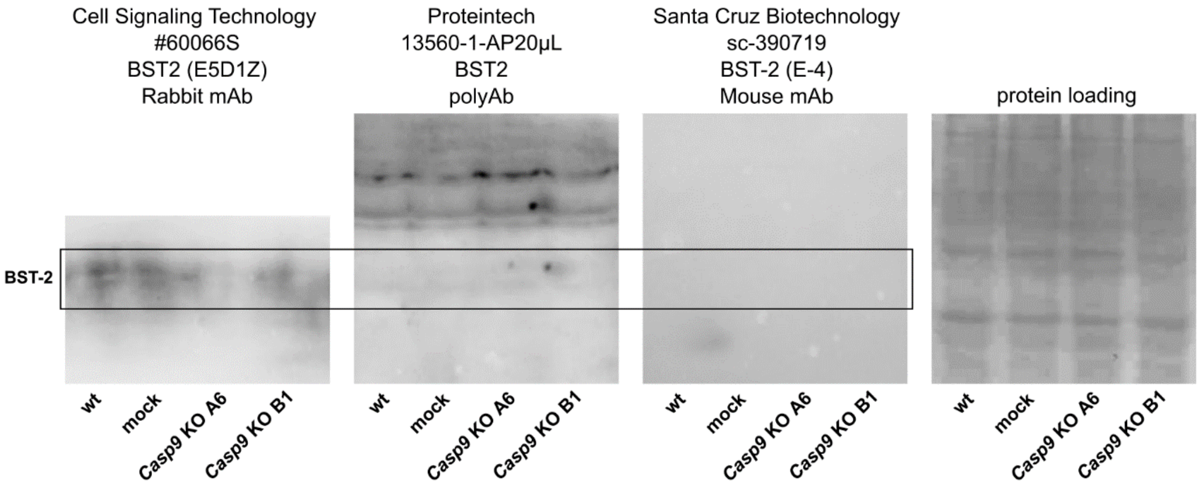

Uncropped western blot images (for Supplementary Data 12)

Cell Signaling Technology #60066S, BST2 (E5D1Z), Rabbit mAb

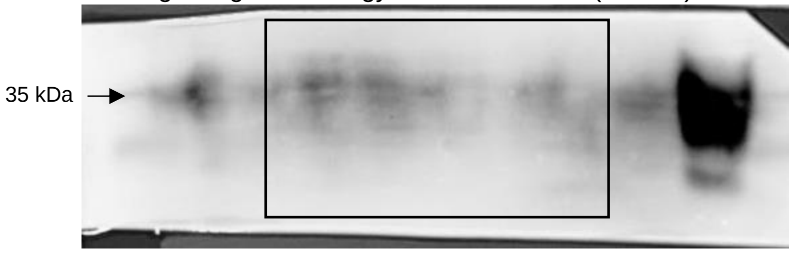

Proteintech 13560-1-AP20μL, BST2, PolyAb

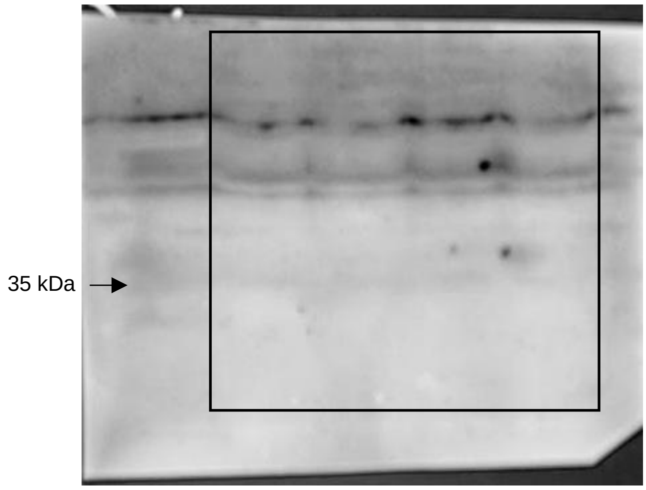

Santa Cruz Biotechnology sc-390719, BST-2 (E-4), Mouse mAb

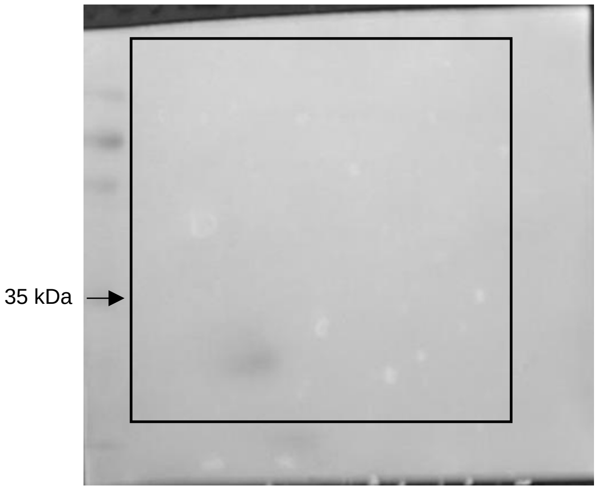

Supplementary Data 13. Uncropped western blot images for Figure 1A

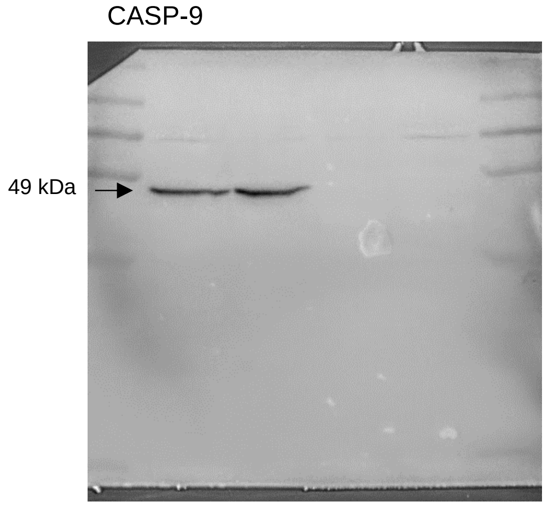

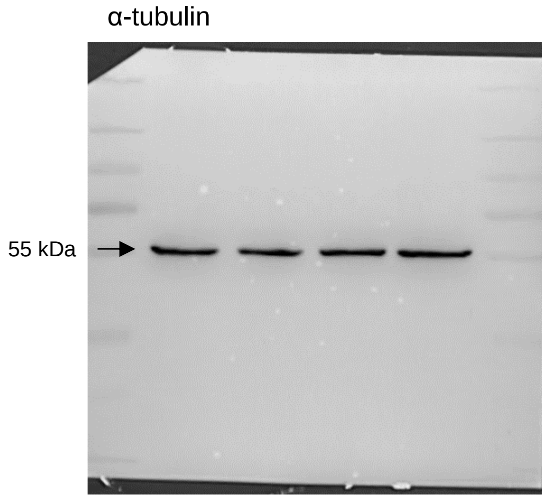

Supplementary Data 14. Uncropped western blot images for Figure 6A

ABHD2

48 kDa →

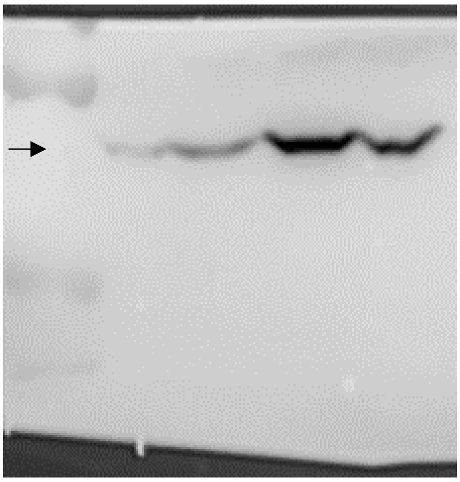

ADAM15

110 kDa (pro form) →

75 kDa (mature) →

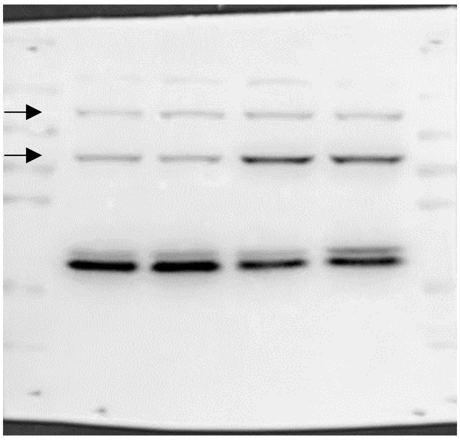

α-tubulin

55 kDa →

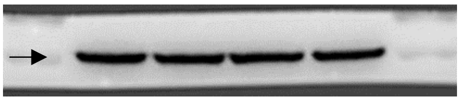

Supplementary Data 15. Uncropped western blot images for Figure 7 (CASP-9 inhibitor)

ABHD2

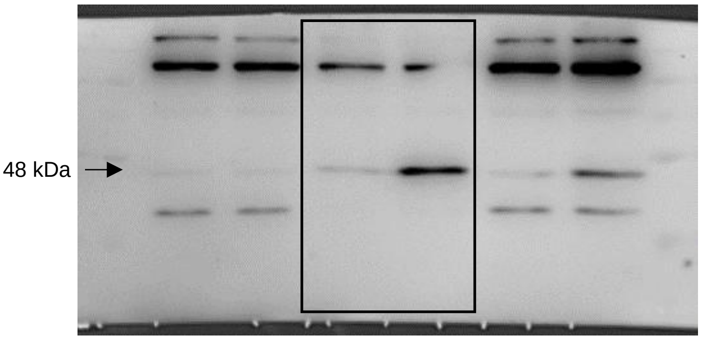

ADAM15

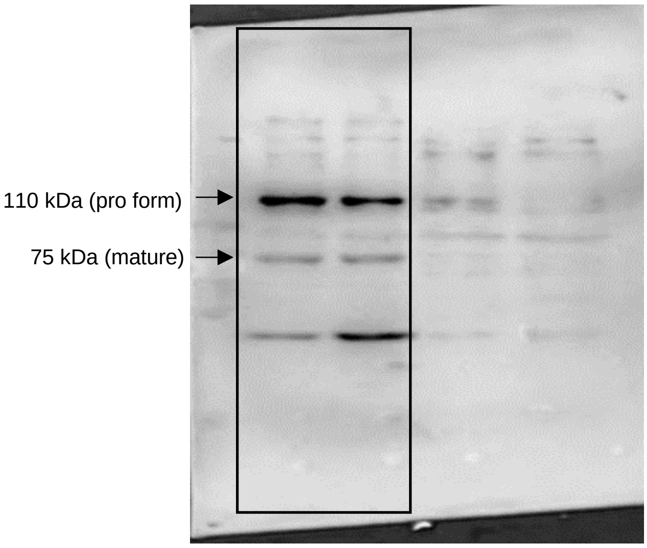

α-tubulin

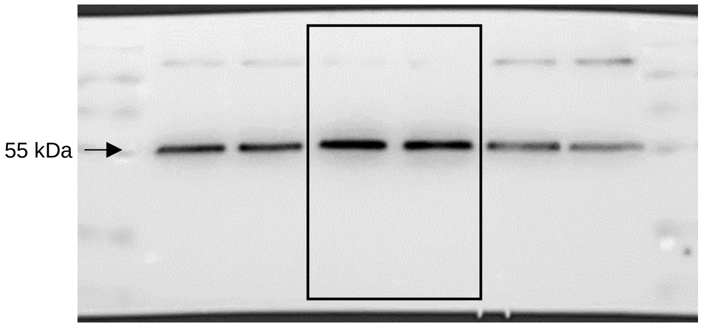

Supplementary Data 16. Uncropped western blot images for Figure 7 (CASP-3/-7 inhibitor)

ABHD2

48 kDa →

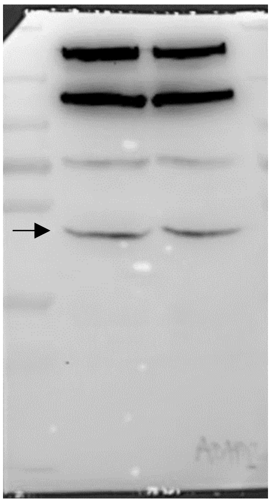

ADAM15

110 kDa (pro form) →

75 kDa (mature) →

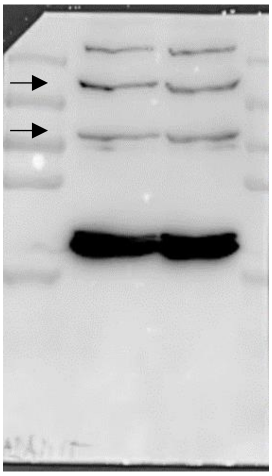

α-tubulin

55 kDa →

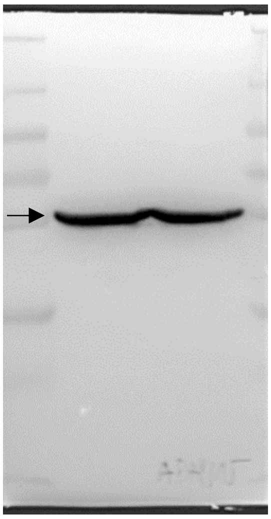

Supplement: Supplementary file 2 — pr3c00641_si_002.pdf [file pr3c00641_si_002.pdf]
